# Values, belief systems and mental health stigma – a scoping review and synthesis of quantitative evidence

**DOI:** 10.1186/s12888-026-08112-y

**Published:** 2026-05-19

**Authors:** Johanna Leona Kummetat, Karsten Valerius, Hannah Jesse, Georg Schomerus, Sven Speerforck

**Affiliations:** 1 Department of Psychiatry and Psychotherapy, Medical Faculty, University Leipzig, Semmelweisstraße 10, 04103 Leipzig, Germany; 2 Department of Psychiatry and Psychotherapy, University of Leipzig Medical Center, Leipzig, Germany; 3 Department of Education and Psychology, Division Health Psychology, Freie Universität Berlin, Berlin, Germany

**Keywords:** Values, Stigma, Mental health, Context, Culture, Review

## Abstract

**Background:**

Stigma related to individuals with mental health problems is a relevant global public health issue. Values, as guiding principles, influence attitudes and behaviors, thus potentially informing the social functions of stigma. *Aim:* To provide a comprehensive overview and synthesis of quantitative evidence on values and belief systems studied in association with mental health stigma. Preregisteration in PROSPERO.

**Methods:**

To match conceptual vagueness and provide a starting point for future research, a scoping review was performed. A systematic search across multiple databases without publication year restrictions was performed, and the results were synthesized according to the PRISMA-ScR guidelines and the Peer Review of Electronic Search Strategies checklist. A quality appraisal was conducted as per adapted Cochrane Collaborations´ suggested inclusions.

**Results:**

A total of 49 original studies published in English or German were included after full-text screening and synthesized into five value clusters: cultural, political, religious/spiritual, personal and milieu-specific dimensions. Across studies, we observed a heterogeneous operationalization of value dimensions, which complicated comparability and synthesis. Overarching trends are illustrated graphically; e.g. conservative values are consistently associated with greater stigma, although they must be read as preliminary and do not allow for causal explanations.

**Conclusion:**

There is a growing interest in contextual aspects such as values studied in association with stigma towards people living with mental health problems. Future research should focus on refining conceptual frameworks and validating value-sensitive approaches that help fuel a deeper understanding of the contextuality of stigma functions.

**Supplementary Information:**

The online version contains supplementary material available at 10.1186/s12888-026-08112-y.

## Introduction

Stigma toward people experiencing mental health problems or who are diagnosed with mental disorders is understood as a complex social phenomenon that follows social functions and can be described in a sequential manner. The stigma process starts by attributing negative stereotypes to a labelled characteristic, followed by negative emotions and discriminatory behaviors directed at individuals who carry the labelled feature [1]. *Stigma,* as we will refer to hereafter, affects individuals’ lives in many detrimental ways, as a barrier to the initial disclosure of a mental health problem or the use of mental health care services, to avoid being labelled and experiencing involuntary social isolation. On a societal level, stigma is discussed as a fundamental cause of perpetuated inequalities and the maintenances of power structure [2]. While prior research has explored the structural, individual, and cultural dimensions of mental health stigma, limited attention has been given to the role of values and belief systems in understanding stigma as a context-informed social mechanism. As Schwartz’s theory of basic human values prominently suggests, values are “desirable, trans-situational goals, varying in importance, that serve as guiding principles in people’s lives” [3]. In the conceptualization of Yang et al. (2019), stigma can be understood as a reaction charged by socialization and personal value systems, which is elicited when an individual perceives a threat to most salient or central values [4]. This perspective highlights that stigmatizing reactions are not merely cognitive or affective but might also be shaped by the evaluator’s subjective belief systems and the perceived jeopardy of values considered central or nonnegotiable. Accordingly, “what matters most” refers to the motivation to either accept or reject an individual living with a mental health problem (including themselves) to protect core values within a local group [5]. The condensed model of cultural elements has recently proposed a parsimonious framework with validity across countries [6]. However, its scope is limited to broad societal-level constructs and does not capture the heterogeneity of more granular conceptualizations studied. For this reason, we did not adopt the model as a guiding theoretical basis but instead chose a more exploratory approach that allows discovering diverse operationalizations of values dimensions studied in stigma research as a first step toward integration. Blind spots of stigma research have been discussed previously [7, 8], highlighting the need for research to foster a deeper understanding of how specific value dimensions are studied in the context of stigma towards people with mental health problems. Schomerus and Angermeyer pinpoint that unless the scientific community critically acknowledges the shortcomings of researchers’ own positionality, including belief systems and perspectives, value dimensions and “potential manifestations of stigma within a liberal cosmopolitan culture” could be overlooked [7]. This has been underlined by the empirical evidence of three studies finding that individuals employing both conservative and liberal values are willing to dislike, intolerate and discriminate others when they perceive ideological dissimilarity, e.g., individuals who violate or possibly threaten their own values and belief systems [9]. Although the importance of examining the role of personal values in shaping mental health stigma has been recognized, to date, only one validated measure, the *value-based stigma inventory* (VASI), enables systematic empirical evaluation, including both modern and liberal dimensions [10]. To the best of our knowledge, no systematic overview of peer-reviewed studies has mapped the quantitative evidence regardingthe associations among value dimensions, belief systems and mental health-related stigma. Previous reviews have addressed related areas but have employed narrower foci: previous studies have investigated multiple levels of attitudes and value dimensions in relation to stigma; other research has limited results to qualitative evidence on male experiences of mental illness stigma [11] or touching culture-informed aspects of stigma among specific minority populations within the USA [12]. Our contribution aims to address the remaining knowledge gap by synthesizing quantitative evidence on overarching value-stigma mechanisms. The objective of the present work is to provide a comprehensive overview of value conceptualizations studied in the field of mental health stigma without limiting this overview to a Western perspective. Hence, the present synthesis aims to contribute to revealing stigma mechanisms in light of their contextual embeddedness, considering macrolevel (cultural context), mesolevel (milieu specificity) and microlevel (individual belief systems) correlates of stigma. At best, the present work can guide future research agendas and aid value-sensitive approaches as well as praxis interventions to address mental health stigma and its detrimental outcomes. The present contribution is guided by the following research questions:

- How are specific personal value dimensions associated with stigmatizing attitudes toward people with mental illness?
- How are specific personal value dimensions associated with stigmatizing attitudes toward help-seeking for a mental health problem?

## Materials and Methods

This review was preregistered in PROSPERO in 2020 (ID CRD42020161859) as a systematic review with the aim of examining how specific personal value dimensions are associated with stigmatizing attitudes towards (a) people with mental illness and (b) help-seeking for mental health problems (for details, please refer to supplementary material S1). Furthermore, we aimed to conduct a meta-analysis if the evidence base allowed. Following an initial systematic literature search and extensive screening of literature, however, it became evident that the existing body of research is highly heterogeneous and, at this stage, does not allow for a meaningful synthesis of associations or meta-analysis. For this reason, we deviated from the initial PROSPERO registration and, in line with best practice recommendations, reframed the approach to conduct a scoping review. Scoping reviews allow unique mapping of evidence by discovering the range of material available, synthesizing and disseminating existing knowledge, identifying gaps and developing further perspectives [13]. This approach enables us to provide a comprehensive overview of the field, which appears in an early, fragmented stage, and to map existing evidence as a necessary foundation for more targeted systematic reviews or meta-analyses. The reporting follows the Preferred Reporting Items for Systematic Reviews and Meta-Analyses extension for Scoping Reviews (PRISMA-ScR; checklist available in the supplementary materials (S6)) [14]. We adopted the framework for scoping reviews by Arksey and O´Malley and followed the suggested stepwise approach: (1) defining and identifying the research question; (2) identifying relevant publications; (3) selecting studies; (4) charting the data; and (5) summarizing, synthesizing and reporting the results [13]. Our principal aim was to map the literature, thereby prioritizing breadth over methodological appraisal Although a formal quality appraisal is not a standard requirement for scoping reviews, it can provide valuable information regarding the strength and reliability of the evidence and was performed as reported below.

### Identifying relevant studies and search strategy

Preliminary search terms were formulated collaboratively by the authors and colleagues from the research team with expertise in the field. The Peer Review of Electronic Search Strategies (PRESS) guideline was used to review the search strategy and topics. The search was conducted via English and German search terms without geographic restrictions or limits on the date of publication. The database search was conducted via EBSCOhost and PubMed (which provides access primarily to the MEDLINE database, along with additional content such as ahead-of-print and non-MEDLINE records), including APA PsycArticles, Psychology and Behavioural Science Collection, APA PsychInfo, and PSYNDEX Literature with PSYNDEX Tests. In the PROSPERO preregistration, both stigmatizing attitudes and help-seeking attitudes were specified as outcomes of interest. During the database searches, however, we observed that including “help-seeking” as a separate search term generated very broad results with limited specificity, often extending far beyond the scope of our research questions. We therefore decided to omit “help-seeking” from the Boolean search string while assuming that studies relevant to our second research question would nevertheless be captured, as research on help-seeking attitudes in the context of mental health stigma almost invariably employs stigma-related terminology. A search strategy for title, abstract and keyword search was developed by combining three main topics, namely, stigma/stigmatizing attitudes, values/social milieus and mental health and illness, utilizing Boolean operators. For mental illness, the search contained both general terms (e.g., “mental disorder*” OR “psychiatric illness”) and specific prevalent disorders (e.g., depress* OR schizophren* OR alcohol*). For details, please see the supplementary material (S2).

Because the databases used differ in their search interfaces, we adapted the syntax accordingly. To maximize consistency and reproducibility, we applied the final Boolean search strings to the “Title” and “Abstract” fields of each database (rather than to all available fields), which ensured that only records with a clear topical focus were retrieved. While this approach reduced the total number of hits, it increased the specificity of the search results. Additional searches were conducted via author correspondence and by reviewing reference lists of previously included articles. The initial search was conducted in January 2020 and updated under the same criteria in 2024, with the last inclusion criterion being 18.08.2024.

### Selection of sources of evidence

Predefined eligibility criteria were applied in accordance with the PICO framework. Population: general population and different subpopulations, e.g., people in treatment, relatives, professionals, without age restrictions. Intervention: Studies that report associations between values and the outcomes mentioned below, e.g. reporting correlational relationships between transparently operationalized personal values and stigma measures (desire for social distance, stereotypes (e.g., dangerousness, blame), emotions (e.g., fear, anger) or attitudes towards help-seeking for a mental health problem, as well as utilization, respectively). Comparison: Other dimensions of values. Outcome: Studies that report (1) stigma towards people with mental illness, including emotional reactions, attitudes, self-devaluation, help-seeking intention or history, or prejudices towards or stereotypes of people with mental illness or specific disorders and correlation with (2) values or belief systems. Types of studies included: All original research articles published in scientific journals, including retrospective, case‒control, cohort, and prospective studies, that report correlational relationships between value or value‒equivalent dimensions and stigma. Studies were limited to quantitative data assessment but not limited to a specific design, such as experimental or group comparisons. Furthermore, publication in a peer-reviewed journal was mandatory. Articles were excluded because they did not meet the inclusion criteria (1 and 2) and because they met the following criteria: (3) did not contain primary data (e.g., reviews, books, comments); (4) were unpublished articles; (5) did not refer to human samples; or (6) were written in languages other than English or German. Although socioculturally constructed roles can have relevant effects on how mental health problems are dealt with, we did not explicitly include gender role socialization (e.g., masculinity and femininity norms) for evaluation. Other research has dealt with this extensively (e.g., a recent scoping review [11] and a WHO technical report [15])). Furthermore, we excluded studies that evaluated differences between ethnic groups solely by typology based on skin color (e.g., Black vs. Caucasian participants) without reporting any value measure or belief systems, as such would imply unnecessary simplification, as detailed elsewhere [16]. To maintain conceptual clarity and in order to avoid unnecessary broadening focus of the scope and shift away from how underlying belief systems and values and limit, interventional studies were excluded in light of the research aim was mapping evidence. While studies evaluating interventions targeted at stigma-related enpoints constitute a substantial but divergent body of literature, which has been addressed in several dedicated systematic reviews and meta-analyses before, interventional study designs were excluded.

All eligible articles published before 18.08.2024 were imported into Citavi6 (Swiss Academic Software GmbH). Most duplicates were removed automatically during the merging of the databases, and the remaining duplicates were manually excluded. The selection of articles was performed in a sequential manner on the basis of the established eligibility criteria described above. Title screening and abstract screening were carried out by three reviewers (KV, HJe, JLK), each of whom examined a distinct part of the search results. The remaining articles were subjected to full-text screening (KV, HJe, JLK). To increase interrater reliability, unclear cases were discussed among four authors (KV, HJe, JK, SvS) until a consensus was reached.

### Data extraction and coding

After the final article selection, detailed information was condensed for each source included (Table 3). The following characteristics were extracted: authors, year of publication, country, sample population, sample size, methodology, type of stigma, value type and assessment, general findings and conclusions. For further extracted study characteristics with detailed measures of both values and mental illness concepts, refer to the supplementary material (S3).

### Quality assessment

A formal quality assessment was conducted for included sources of evidence. Six quality criteria as per Cochrane Collaborations´ suggestions were assessed during data extraction This framework has been frequently adapted for systematic and scoping reviews addressing complex public health topics. For the present work, we have applied six pragmatic quality criteria adapted from recent applications of this framework in stigma-related evidence synthesis (Majeed, et al., 2024; Evans-Polce, et al., 2015): (i) aims clearly stated, (ii) design appropriate to stated objectives, (iii) justification given for sample size, (iv) evidence provided of reliability or validity of measures used, (v) statistics accurately reported, and (vi) sample selection was relatively unbiased. Two raters (HJe, JLK) performed the quality assessment independently; deviations in assessments were discussed.

### Analytic strategy

Since the conceptualization and operationalization of values and belief systems widely varied across the included sources, studies did not show sufficient homogeneity for aggregation into meta-analyses. Hence, the prevalence of concepts and operationalization used in the reviewed research were synthesized narratively and provide more detailed insight into how values and belief systems have been studied in association with mental illness stigma.

To organize the narrative synthesis of results in a comprehensive manner, clusters of value dimensions were determined. Records were screened and inductively categorized into content-related clusters during the screening phase. These inductive clusters were derived from operationalizations and measures used in the sources, which are provided in the supplementary material (S4). Clusters were discussed and modified iteratively with peer researchers in the working group. Value dimensions were assigned abductively, e.g., in the sense of identifying the most plausible approach for the material and research question. Following a peer-based methodology, controversial aspects were addressed through discussion to reach consensus.

## Results

**Fig. 1:**
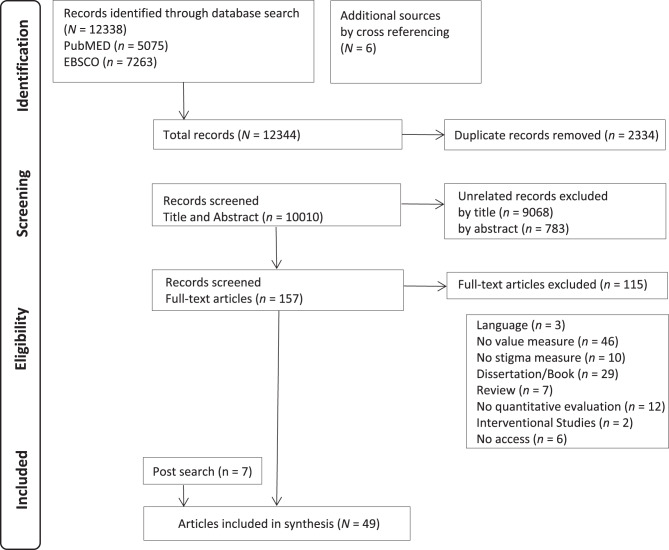
PRISMA flow chart of the study selection process

After a systematic search of five literature databases (PubMed, EBSCO, MEDLINE, PsychINFO and Embase), the initial search yielded 12,338 records. Six additional records were drawn from the reference lists, and the authors were contacted. As shown in Fig. 1, 10,010 records remained for analysis after removing duplicates. *N* = 157 full texts were assessed for eligibility, with 42 unique articles included in the final review following full-text screening. *N* = 7 records were added after the database search by expert consultation and cross-reference checking, leading to 49 studies that were included in the review (Fig. 1).

In the following sections, key findings of the reviewed sources of evidence are summarized descriptively. The narrative synthesis involves value dimensions and overarching conceptualizations, identified outcome measures, methodological approaches and a synopsis of results.

### Study description and characteristics

**Fig. 2:**
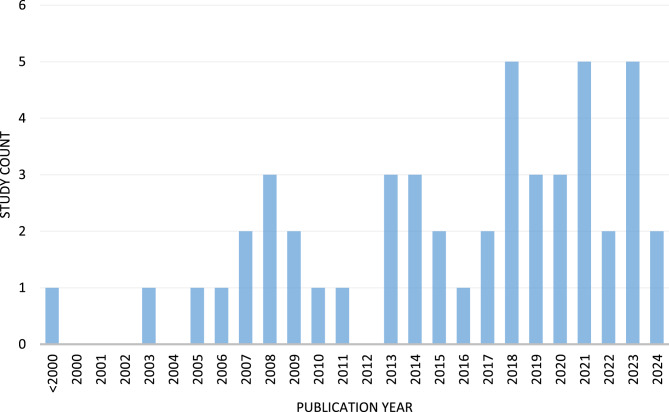
Numbers of publications per Year

We identified a total of *N* = 49 unique articles published between 1997 and 18.08.2024. For the number of publications by year, see Fig. 2.

A total of 83.7% (*n* = 41) of the sources focused on a single study population, whereas 16.3% (*n* = 8) analysed populations in multiple countries or studied multiple subsamples by geographical or cultural background. Publications included studied samples recruited from North America (*n* = 34) including Canada (*n* = 4) and Mexico (*n* = 1), Europe (*n* = 10 including Germany *n* = 4), the United Kingdom of Great Britain (UK) *n* = 2, each *n* = 1 sample from Norway, Sweden, Greece and France), Asia (*n* = 7 including China, Taiwan, India, Israel, Lebanon, Turkey), Australia (*n* = 5), and South America with *n* = 1 sample from Columbia and Africa, with *n* = 1 sample from Ghana. However, the geographic location of sampling does not determine ethnic or cultural background specificities in various cases [17, 18]. Most of the study samples were drawn from university students (*n* = 25) and the general population (*n* = 17); few focused on practitioners and professionals (*n* = 2), high school students (*n* = 1), young people (*n* = 1) and older people (*n* = 2) and subgroups of community members, i.e., adults with psychiatric conditions (*n* = 2) and their relatives (*n* = 1). Some sources restricted their sample to residents of rural areas [19, 20]. All included records are referenced and described by their main characteristics and findings in Table 3. A more detailed description of the included studies is provided in the supplementary table (S3).

### Study design and methodological approaches

Overall, all the sources included applied a cross-sectional nonexperimental design, focusing on the associations between values and measures of stigma (*N =* 49). The included studies used online (*n* = 44) or face-to-face (*n* = 5) survey modes [18, 21, 22]. For *n* = 16 sources, vignettes or videos were used as stimuli. *N* = 23 sources used measures linked with help-seeking for mental health conditions, including attitudes towards receiving professional help, intentions to seek help and treatment experience. Some sources have evaluated between-group comparisons, including different types of values [23], different spatial [24] or cultural backgrounds (i.e. [17, 25]), or comparisons of different subsamples, i.e., community members with and without psychiatric disabilities [22, 26], as well as populations of professional background vs. the general population [27] or by ethnicity [28]. Value dimensions were mostly assessed as latent measures by self-report questionnaires in which participants indicated predefined culturally specific positions [29–34], sometimes alongside spatial indicators such as neighborhood density [22], or intertwined with the prevalent state of economic development [35]. Furthermore, religious, ideological or context-informed beliefs [36, 37] and political attitudes [38] have been studied in association with mental health stigma and help-seeking outcomes.

#### Quality assessment

Eighty nine percent of studies *(n* = 44) met either four (*n* = 28) or more (*n* = 17) of the six assessment criteria (Table 1). However, none of the studies met all six criteria with most studies lacking quality in generalizability of samples as mostly selective samples were drawn from university students. No studies were excluded due to their quality. Detailed quality rating per study can be found in supplements (S5).

**Table 1:** Formal quality assessment of included studies (*N* = 49)

	Quality criteria	Yes	No
		n (%)	n (%)
1	Were aims clearly stated?	49 (100%)	0 (0%)
2	Was design appropriate for the stated objectives?	49 (100%)	0 (0%)
3	Was a justification for sample size given?	17 (34,7%)	32 (65,3%)
4	Was evidence on reliability and validity of measures provided?	44 (89,8%)	5 (10,2%)
5	Were statistics reported accurately?	49 (100%)	0 (0%)
6	Was the sample selection relatively unbiased?	1 (2,0%)	48 (98,0%)

### Stigma outcomes

Both between and within value clusters, outcome assessment differed in operationalization and methodology. Outcomes mostly assess public stigma through self-reported attitudes towards people with mental illness. Most predominantly applied were self-reported desires for and actual choices of social distance toward people with mental illness (*n* = 8), as well as self-reported or perceived public attitudes, judgments, emotional reactions and behavioural intentions. Furthermore, help-seeking intentions (*n* = 11) or help-seeking in personal history (*n* = 5) were assessed. The most prominent stigma targets were defined as people living with mental illness without diagnostic specification (*n* = 29). When specified, the diagnoses of mental health conditions used were mostly depression (*n* = 13), schizophrenia (*n* = 12), alcohol (*n* = 3) or substance use disorder (*n* = 2) and anxiety (*n* = 3). The majority of the included studies assessed stigma as reactions to a labelled condition.

#### Attitudes and reactions toward people living with mental illness

##### Judgements of responsibility and deservingness

The attribution of responsibility for one’s own mental health condition emerged as a notable aspect of the attitudinal stigma dimension, operationalized in various ways across the included studies, some more explicitly others rather implicitly: the attribution of responsibility was mostly assessed through self-reports, some with emphasis on personal agency and etiology beliefs [26, 39, 40], while one also used the Brief Implicit Association Test to assess guilt-related stereotypes about mental illness [26]. Another study included recovery-oriented attitudes—i.e., the belief that people can regain agency and live a fulfilled life despite experiencing crises—as an inverse indicator of community stigma [22]. The attribution of responsibility has also been linked with judgments of deservingness, e.g., whether individuals with substance use disorders should receive high- or low-quality care [41].

##### Emotional reactions

Perceived dangerousness was assessed in five studies. Additionally, negative (e.g.unpleasant emotional reactions, anger and disappointment) [42] and positive affect (sympathy, concern) are intermediate variables linking value endorsement to diservingness judgments and satisfaction with the quality of care in substance-abuse health services [41].

##### Desire for social distance

The desire for social distance measures behavioural intention at the end of a stigma process that is consecutive to labelling, stereotyping and negative affect [43]. The stigma measures mostly involve the established 7-item version of the Link et al. Social Distance Scale, *n* = 6 [44]. One source used a depression-specific version of the 12-Item Social Distance Scale [45]. One included study approached social distance as an actual behavioural measure by reporting individuals’ choice of seating distance [46].

##### Behavioural discrimination and microaggressions

Stigma operationalization further included behavioural reactions by measuring subtle forms of discrimination, more specifically, the expression of microaggressions [22, 42, 47], as well as everyday discrimination experiences [48]. One study evaluated respondents’ willingness to allocate donations to honor individuals who sustained somatic versus psychological impairments [49].

#### Levels of stigma

Stigma operates on multiple interconnected levels, influencing both individual experiences and societal structures [43, 50–52], with the growing relevance of social media to the framing of mental health conditions through public disclosure of experiences on such platforms [53, 54]. The literature in the field of stigma research commonly distinguishes between stigma at the structural, interpersonal and individual levels [55, 56]. Among the included sources, we found articles pertaining to structural stigma, public stigma, perceived stigma, self-stigma and stigma by affiliation, as exemplified below:

##### Structural stigma

The organization of institutional health care, policies and the allocation of resources can perpetuate stigma by limiting access to fair access to care for individuals living with treatable conditions. One study assessed the clinically relevant component of structural stigma by biased treatment recommendations employed by professional staff in mental health care [41]. Another source examined structural disadvantages for individuals with mental health by comparing respondents´ readiness to award military honors and to donate to veterans with psychological versus psychological impairments [49]. Findings indicated structural stigma as reflected in greater willingness to award honors and provide financial support for those with physical injuries.

##### Public stigma

Narratives that exist in general society perpetuate framings of publicly perceived “in-groups” and create thresholds of what is either accepted or socially sanctioned, exemplified by the expression of social distance. Public stigma has been evaluated by measures including, i.e., willingness to live or work with someone with mental health conditions, spending an evening with or to accept them as members of one family through marriage [57]. However, most commonly such level of stigma has been assessed by referring to desire for social distance [21, 30, 58]. One study assessed public stigma as a response to diagnostic labels rather than behaviour descriptions [23].

##### Perceived stigma

Other articles included perceived social norms [37] and anticipation of stigma by means of personal concers that the utilization of help for mental health care could harm their reputation [59]. One source evaluated the subjectively perceived social support by friends, family and significant others [18].

##### Self-stigma

When perceived stigmatizing public attitudes are aware, approved and applied to oneself, negative evaluation is internalized as self-stigma. One included article compared attitudes towards people living with mental illness in the general public with the attitudes of people living with mental health conditions and the degree of application of negative stereotypes to themselves [26]. Such measures of self-stigma further encompass anticipated concerns about disclosure [28, 60, 61], which may affect help-seeking and engagement with mental health services.

### Stigma by affiliation

Family members and next of kin to individuals in mental health crises are underestimated and unpaid resources for recovery. However, such informal caregivers face burdens on multiple levels themselves, including stigma from affiliation. The studies included in our contribution have assessed stigma by affiliation directly [39] and as belief that depression brings shame to the family as an anticipation of stigma [30].

#### Help-seeking

The operationalizations associated with help-seeking were expectations of disclosure of having mental illness experience [62]and perceived barriers to help-seeking [63]. Löve and colleagues assessed the willingness to disclose about depression experiences as a secondary outcome by self-reporting agreement with the statement “If I had depression, I wouldn´t tell” [40]. Intentions to seek professional help [18, 24, 29, 32, 33, 61, 62, 64] and health care utilization and experience with having sought professional counselling [18, 20] were assessed as consequences of perceived [48] and (internalized) self-stigma [28, 59, 61].

### Conceptualization of value dimensions and belief systems

The studies examined a broad range of values and milieu-specific beliefs, combining a heterogeneous picture of correlates of stigma towards people with mental illness. Owing to the indistinct conceptualization and application of value dimensions across the field of study, overlapping across and heterogeneity within the presented groupings is expected to some extent. To provide an overview of prevalent topics, the included studies are thematically organized by their most prominent topic within clusters. An integrated overview of abductive clusters of value dimensions is provided in Table 2, including key methodological features as well as example measures used within included studies. Given the heterogeneity of the evidence, this synthesis can at best be preliminary description. For more detailed information on measures used, please refer to supplementary material (S4).

**Table 2:** Abductive clusters of value dimensions and synthesis

Value Cluster	Description	Examples	Operationalization & Instruments	Tendency of Associations
**Cultural**	Values, lifestyles in different cultural or geographical environments.	Collectivism vs. individualism; Long-term orientation; Cultural belief systems (e.g., as compared between Turkish, Spanish vs. German samples).	Asian Values Scale (AVS) 36-item (Kim et al. 2005) Vertical-Horizontal Individualism-Collectivism Scale (VHIC, 16-item) (Triandis und Gelfand 1998) Mexican American Cultural Values Scale (MACVS) (Knight et al. 2010)	Interdependent orientation, face concern, Long-term orientation associated with ‘wait-and-see’ attitude.
**Religious/** **Spiritual**	Values and life orientations connected to religious affiliation or spiritual beliefs.	Belief in prayer to heal symptoms; Christian Orthodoxy; Religious Fundamentalism.	Religious Beliefs about MI −16 (Wesselmann & Graziano, 2010) Items on prayer and divine healing The Christian Orthodoxy Scale (Hunsberger, 1989)	Orthodoxy associated with higher stigma, less openness to professional help-seeking, believe of divine healing linked to more reliance on spiritual solutions over medical treatment. In traditional religious Muslim community: strong emotional support from family associated with more openness.
**Political**	Socio-political attitudes and normative worldviews.	Right-Wing Authoritarianism, Social Dominance Orientation, Belief in a Just World, Dangerous World Beliefs	Social Dominance Orientation (SDO) − 12 items (Pratto, et al., 1994) Right-Wing Authoritarianism (RWA) Scale − 20 items (Altemeyer, 1981)	Consistently associated with higher stigma and greater social distance toward individuals living with mental illness.
**Personal**	Individual- values assessed with standardized value surveys.	Schwartz Value Scale, Portrait Value Questionnaire Portrait Values Questionnaire; Schwartz Value Survey; Measures of self-transcendence vs. self-enhancement.	Schwartz Value Survey, 57-item (Schwartz. 1992) Portrait Value Questionnaire (PVQ), 57-items (Schwartz &Bardi, 2001)	Self-transcendence (e.g., benevolence, universalism) linked to lower stigma; Self-enhancement (e.g., power, achievement) linked to higher stigma.
**Milieu-Specific**	Composite belief systems shaped by spatial/geographic context and sociodemographic factors.	Composite “suburban values”; Honor ideologies.	Composite “suburban values” (dense neighbourhoods by ZIP code and conservation values) (Gonzales et al. 2018) Liverpool Stoicism Scale (LSS), 20-item (Hagger & Chatzisarantis, 2009) Honor Ideology of Manhood (HIM-16) (Barnes, 2012).	Rural/agrarian milieus associated with more traditional views and less openness to help-seeking. Honor ideology linked to higher concern that seeking help for mental health problems indicate personal weakness and harm reputations.

*Note.* For detailed description of operationalization applied within value clusters and original sources of measures used, please refer to supplementary material (S4)

#### Cultural values

Several studies have analysed cultural differences alongside attitudes toward mental health problems and service utilization. This broad cluster covers various perspectives on stigma, including intra- and interculturally located methodologies. The evaluation of cultural influences in the help-seeking process has mostly included comparisons between geographic regions: Bermejo and colleagues (2014) compared attitudes towards the use of psychotherapeutic and psychiatric services in Germany, Turkey, and Spain. Spanish and Turkish respondents reported more positive attitudes towards help-seeking than German respondents did, although Turkish participants simultaneously expressed less openness to talking about psychological problems. Across the sample, the endorsement of long-term orientation values was associated with a more reserved “wait-and-see” attitude towards seeking professional help. In addition, female gender, older age, adherence to traditional gender role attitudes, lower self-reported psychological distress, and more collectivist orientations were linked to more favourable attitudes toward the use of psychiatric and psychotherapeutic services.

Cross-cultural studies consistently highlight the influence of cultural values and social norms on mental health stigma. For example, Gillespie-Lynch et al. [23] compared stigma across multiple conditions in student samples from the U.S. and Lebanon, finding no between-country differences but showing that greater stigma co-occurred with greater acceptance of inequality, less openness, and lower cognitive empathy. Notably, diagnostic labels were stigmatized less than behaviors were. A prominent framework in the field is face concern, the need to maintain a culturally salient social image, which is particularly strong in East Asian contexts [63, 65]. In a study of students from Hong Kong, China, and the U.S., both groups of Chinese individuals reported greater stigma and more barriers to help-seeking than European Americans did, with concern of losing face mediating these cultural differences [63]. Similarly, greater depression stigma among Asian Canadians than among European Canadians has been reported [30], and mediators, including familial shame, perceived social norms, conservative values, and social dominance orientation, of which familial shame and norms have the strongest associations [28]. Papadopoulos et al. [31] examined leaning towards individualism versus collectivism across UK-based samples (White-English, American, Greek/Greek Cypriot, Chinese). Americans reported the highest individualism and lowest stigma, whereas Chinese participants reported the highest collectivism and highest stigma. Stigma constructs (social restrictiveness, low benevolence) were found to be more prevalent among individuals employing collectivist orientations and, additionally, with lower mental health knowledge and shorter residences in the UK. Together, these findings indicate that stigma is shaped not only by cultural context but also by co-occuring specific value orientations and socially shared norms such as collectivist and face concerns.

##### Bicultural aspects and cultural socialization

The endorsement of values has been explored in light of specific imprints by cultural context. It has further been assessed within subpopulations, i.e., people who moved from one geographic region to another. Thus, another prominent focus is the level of *enculturation* (adherence to cultural values of origin) and *acculturation* (adherence to host cultural values) [66] and how such values can inform mental health stigma [17, 67]. Research consistently links adherence to traditional cultural values with less favourable help-seeking attitudes across diverse ethnic groups. Among Asian Americans, stronger enculturation to “Asian” value dimensions such as emotional self-control and conformity to norms as “barriers for help-seeking within Asian Americans as such they pronounce the value of exercising restraint of emotions—especially when experiencing difficult ones” was associated with less willingness to seek professional help, independent of demographics and acculturation status [29, 68]. While some studies have shown that acculturation to “European American values” is unrelated to help-seeking [68], others have reported negative associations in specific contexts, suggesting that bicultural competence rather than simple acculturation may promote more adaptive outcomes [34]. Lower adherence to Asian values, female gender, and lower stigma were further indicative of more attitudinal readiness to utilize treatment [33]. Evidence from immigrant samples underscores the role of heritage. For example, Chinese immigrants in Canada endorsed more negative help-seeking attitudes than community samples did, with higher endorsement of traditional beliefs corresponding to stigma, whereas perceived social support fuelled more favourable attitudes towards help-seeking [18]. Similarly, Australian-born Chinese individuals presented lower stigma than Chinese immigrant or Taiwanese respondents did, with cultural practices linked mainly to perceptions of dangerousness [69]. A comparative study revealed that traditional Asian values are associated with greater onset responsibility and courtesy stigma, moderated by ethnic background [39]. In addition to Asian populations, studies of Mexican Americans have demonstrated that familism and traditional gender roles are negatively related to the utilization of formal mental health care [70], whereas younger generation status and adherence to Mexican values predict greater willingness to consult a counselor, independent of acculturation to American values [32]. Ethnocultural gender roles further differentiated attitudes among Latina women, with traditionalism linked to less favourable attitudes and egalitarian orientations linked to greater openness [71]. In Filipino Americans, despite high levels of psychological distress, service utilization remains lower than that in the general population [72, 73]. Colonial mentality (CM), understood as internalized oppression, was shown to shape help-seeking indirectly: higher CM was associated with lower acculturation to European American values and, in turn, with less openness to psychological problems; further strong adherence to Filipino interpersonal norms predicted lower help-seeking and greater stigma concerns [66, 67]. Finally, within a U.S. college student sample, Islamic religiousness and biomedical etiology beliefs predicted greater openness to mental health treatment, whereas greater integration with prevailing dominant culture was associated with greater confidence in services [74]. Taken together, these findings illustrate that collectivist, conservative, and familism value orientations are associated with higher levels of stigma and lower help-seeking, whereas acculturation effects are less consistent and may depend on bicultural integration, religious orientation, and contextual moderators.

##### Shared cultural values

Different scientists in the field have added another perspective to ethnic-comparative research by evaluating the values shared in different subsamples. Similar values of Latinx and Asian cultural groups, for example, were found to influence help-seeking behaviours [75]. Zhou et al. investigated adherence to cultural values within Latinx and Asian college students alongside seeking help [47], denominating two ethnicity-unspecific cultural factors, referred to as Interdependent Orientation, “the importance of one’s family and in-group”, and Cultural Obligation, “the importance of actively participating in and preserving one’s culture around” [47]. However, endorsement of interdependent orientation values was consistently associated with mental health stigma and co-occurred with less perceived need for mental health treatment, regardless of ethnic group; on the other hand, cultural obligation did not predict perceived need- or help-seeking behavior. Conditions described in vignettes in two study populations from New York City (US) and Beirut (Lebanon) that were perceived as more dangerous (e.g., psychopathy) were stigmatized more than those perceived as less dangerous (e.g., autism), with no significant differences in stigma level between the two populations per se [23]. The participants from both Singapore and the USA indicated experiencing strength, self-efficacy, resourcefulness and a recovery orientation when asked about their appraisal of the diagnosed condition, with American respondents indicating significantly more positive aspects of mental health conditions [76]. Additionally, both subsamples reported experiences of stigma within the subscales of discrimination and disclosure. Comparing samples with individuals from France and Greece, Lampropoulis and colleagues concluded that cross-national differences, e.g., in dominance-competitveness and authoritarianism-dangerousness pathways, may reflect not only ethnic-cultural features but also structural features of the health care system [77].

Other studies suggest differences in the perceived need for counselling and lower rates of seeking help from mental health services in Black/African Americans than in European Americans. However, the data of Lannin et al. did not suggest group differences between Black/African and White/European respondents regarding the stigma measure [28].

A language-based approach highlights how culturally informed sets of meaning can be evoked through linguistic framing by investigating the impact of native versus foreign language usage on the acceptance of mental health treatment [78]. Heller et al. demonstrated that bilingual Chinese–English speakers gave stronger treatment recommendations when vignettes were presented in English versus Chinese, with traditional Asian value orientation moderating the effect. Specifically, in the Chinese version, stronger endorsement of Asian values reduced treatment recommendations, underscoring the interplay of language, culture, and stigma [78]. Cultural and linguistic contexts may also shape how mental health conditions are conceptualized and perceived publicly. For example, terms of conditions,e.g. major depression, that are widely recognized and historically influenced public awareness and interpretation of mental health conditions in western countries, are absent or limited in active use in some Asian contexts. Changes of terminology and public discourse over time illustrate how linguistic shifts can mirror social understanding and perception of mental health concepts. In Japan, for example, terminology for major depression diagnosis has only been indicated in recent years.

#### Religious and spirituality-informed values

In articles that evaluated spirituality and its effect on intentions to seek professional counselling, spirituality-informed value dimensions were assessed via self-rated religious affiliation and practice (frequency of church attendance and prayers) as part of “traditional Mexican values” in Mexican American attendees of community colleges [32]. Others have examined religious and traditional cultural beliefs about depression in a sample of Muslim adolescents [37]. The majority of the respondents indicated that reciting from the Koran relieved their mental distress. However, those who believed in prayer to heal depression were less likely to accept a diagnosis (depression). Half of the sample reported the family preference of a Muslim doctor for medical care and wanted a female doctor for female family members. This underlines the relevance of physicians’ cultural acknowledgement and recognition of spiritual beliefs as “many felt that the doctors lack cultural competence to address depression” [37]. Other studies have shown that individual religious cultural values indirectly influence the willingness to seek counselling through etiology beliefs and self-stigma. In a sample of Mexican American college women, the influence of spiritual etiology beliefs (i.e., “caused by deamons” or “punishment for sins”) was associated with reduced help-seeking intentions by the co-occurrence of increased self-stigma, whereas this relationship was not found for individuals employing biological etiology beliefs regarding mental health problems (i.e., “brain disorders” or “hereditary”) [64]. A study with U.S. college students that predominantly reported Christian faiths revealed that religious fundamentalism, but not Christian Orthodoxy, was associated with stigma towards people with schizophrenia [36]. This relationship did not hold regarding stigma towards people with diabetes and a Christian lifestyle (control condition). Additionally, beliefs about mental illness and respondents’ willingness to provide support for a hypothetical friend described as “suffering from depression” and differentiating between *secular*, *instrumental* and *spiritual* support were compared within groups of different Christian affiliations/denominations (i.e., Roman Catholic, evangelist) [79]. The findings indicate that differences in the types of support an individual is willing to provide operate as a function of religious affiliation and beliefs about mental illness. Within a sample of Muslim Americans, Mudryk and Johnson found (islamic) religiousness in combination with biomedical beliefs indicative of a more positive stance towards professional treatment [74]. Additionally, a higher degree of integration with mainstream culture was related to greater confidence in professional mental health care [74]. On the one hand, family cohesion and spirituality are historically rooted resources for many individuals when coping with problems, including mental health issues. On the other hand, researchers reported negative experiences with the Christian church when facing mental illness, including abandonment by institutions, equating mental illness with the work of demons and attributing mental illness to personal responsibility as a result of a sin [80]. Stanford and colleagues also reported that *“women being significantly more likely to have their mental illness dismissed by church and/or be told not to take psychiatric medication*” [80]. Furthermore, lay theories of the etiology and onset responsibility of mental illnesses, such as biogenetic, familial/social, personal (e.g., onset responsibility/religious causes) and religious beliefs, are associated with (a) intentions and willingness to seek help from a counselor [36, 37, 64] and (b) different types of support that respondents are willing to provide to individuals with depression [79].

#### Political values

A body of research has analysed predictors of mental illness stigma, focusing predominantly on conservative political identification. The studies included in this review constructed and analysed various concepts related to political value orientation. These include right-wing authoritarianism, which involves “ideological commitment to tradition, authority, religion and social convention against threats of change and political rebellion” [81]. The associations of six components in the right-wing extremist orientation, e.g., Advocacy of dictatorship, Chauvinism, Xenophobia, Anti-Semitism, Social Darwinism, Trivialization of National Socialism, and the desire for social distance from people with mental illness (alcohol and drug use, schizophrenia, depression), were evaluated [82]. In this study of the German general public, however, right-wing extremism was only marginally related to social distance from mental health conditions. In contrast, various studies have demonstrated a link between conservative, authoritarian attitudes and stigma. Different findings have been described within a U.S. sample: the findings of Altenmeyer and colleagues (1981) indicated that higher levels of right wing authoritarianism are associated with increased stigma [83]. Similarly, another source reported that individuals scoring high on Right Wing Authoritarianism endorsed higher-than-average stigma outcomes toward vignette characters living with depression and schizophrenia within this study; outcomes included desire for social distance, stereotypes, negative attributions as well as affect, perceptions of danger and exclusionary statements [42]. Entangled with Right Wing Authoritarianism are dangerous world beliefs used to assess social worldviews associated with threat, danger and unpredictability as well as perceived threats to the in-group and self [42]. However, dangerous world beliefs were not found to be an influencing factor in relation to stigma in this study. The theory of social dominance addresses the stigma process as a function of maintaining social hierarchies [84], in which “some groups hold high status and others hold low status”. Social dominance orientation predicts prejudice towards mental illness; notably, a less pronounced relationship is observed in a sample of health professionals/trainees than in the general population [27]. The relevance of *dominance-competitiveness and authoritarianism-dangerousness* for stigma towards schizophrenia has been replicated in a comparison of two geographic contexts (France and Greece) [77]. The authors further report different relationships of the pathways: while both pathways are significant in predicting social distance in the French sample, in the Greek sample, stigma towards people with schizophrenia was only predicted by authoritarianism-dangerousness. The authors conclude that geographical differences in sociocognitive processes could indicate “differences in the status of community health service for people with mental disorders” and, in turn, be indicative of a perception of mental disorders mostly in terms of deviance, where advanced integration is lacking [77].

In another study from Germany, the influence of political value orientations on stigma was compared between two groups: first, outpatients from mental health care centers and, second, a control group matched with sociodemographics [26]. The authors expanded on the link between (political) world views and mental illness stigma by including a) the Calvinist concept of protestant (work) ethic, which grasps individuals’ views on self-discipline and hard work as a fundamental cause for wellbeing and success in life, and b) beliefs in a just world, which refers to the view that people obtain what they deserve and deserve what they obtain. In both groups, stigmatizing beliefs are consistently and positively linked to protestant ethics. However, implicit guilt-related stereotypes were only positively associated with protestant ethics among community members, whereas in the group of outpatients, stronger beliefs in a just world for self were related to reduced self-stigma but simultaneously to more implicit blame from people with mental health problems [26]. Within a sample of trained health professionals in Norway, the predictive value of individual differences in sociopolitical attitudes regarding stigma toward vignette-described mental health conditions was compared against a general sample [27]. The authors reported that within their sample, social dominance orientation had the most predictive value for victim blaming, and less empathy was expressed. Beliefs in a just world predicted less empathy for males but not for females with a mental illness, and right-wing authoritarianism was not a significant predictor of any type of prejudice assessed that described different conditions.

#### Personal values

Self-conception and values are guiding principles of individuals’ perceptions of themselves, others and the world that motivate behaviour. Personal values are therefore likely to determine how one perceives and interacts with other people. A variety of personal value dimension studies overlap with the political, spiritual and cultural beliefs and motivations mentioned above [6]. However, we grouped studies according to *personal values* that included either “guiding principles of their own life” (i.e., the Schwartz Values Survey, 57-Item) or references that included concepts of recovery or resourcefulness. This is to mirror the manifold and inconsistent definitions and conceptual facets of values studied in relation to stigma, as accumulated in the present review.

Angermeyer and Matschinger used a German representative population survey, asking respondents to indicate the social desirability of their behavioural patterns [21]. Their maximum-likelihood factor analysis yielded three separate value dimensions that accounted for differences in desire for social distance, with participants indicating more *traditional* (achievement, duty/acceptance, materialism) orientations expressing more social distance toward people with mental health conditions than those with rather *liberal* (equalitly, justice, tolerance) and modern (self-realization, hedonism, postmaterialism) orientations, which reported a lower desire for social distance [21].

Some studies have evaluated mental health care workers’ personal values with respect to potential service implications for recipients. In a sample of Australian nurses, Skinner et al. reported that perceived responsibility for prolonged use of alcohol or heroin increased with greater importance assigned to conservation values (conformity, security, tradition) [41]. Negative affect (anger, disappointment) toward the patient increased with greater importance assigned to conservation values and decreased with greater importance assigned to self-transcendence values, whereas positive affect toward the patient (sympathy, concern) increased with greater importance assigned to self-transcendence (equality, benevolence, universalism) values. Additionally, negative and positive affect had explanatory value for nurses’ judgments of whether high- or low-quality care is desirable. A study conducted in Israel indicated that community workers who preferred self-transcendence values over self-enhancement values demonstrated more positive attitudes toward their clients’ empowerment, a greater sense of similarity, and negative attitudes toward exclusion [85]. These findings suggest that prioritizing values such as benevolence and universalism over achievement and power may contribute to better outcomes in community work. Additionally, self-direction, belonging to openness to change, was positively associated with positive attitudes toward empowerment, a sense of similarity and a negative attitude toward exclusion. Within a sample of medical students, Lannin et al. reported a negative association between help-seeking stigma and rating compassionate values more important than self-interested values such as social power, authority, success, and capability [61]. Compassionate values also had a significant indirect effect via stigma on help-seeking attitudes during distress. In line with these findings, the results of Norman et al. highlight the association between self-transcendence values and lower social distance toward people with schizophrenia and depression, which contrasts with the positive relationship between greater desire for social distance and self-enhancement and conservatism values reported in a sample of Canadian college students [58].

#### Milieu-specific values

##### Honor beliefs

Prior research has examined the role of honor-related cultural norms, defining honor as a set of beliefs and values emphasizing personal reputation, strength, and social image. Individuals living in “honor states” of the U.S., where these values are salient, expressed greater concern that seeking mental health care would signal personal weakness and harm their reputation [59]. Correspondingly, in states with a high prevalence of honor ideology, investment in mental health resources was lower, and parents were less likely to seek services for their children. Other concepts related to on ideologies, e.g., honour of manhood, include aspects of gender role models and attributes such as reputation and weakness. Individuals, who strongly endorse honor-related beliefs “were especially concerned that seeking help for mental health problems would indicate personal weakness and harm their reputations” despite respondents´ gender. In the general population, Schiffer and Saucier studied how masculine honor beliefs (e.g., that aggression is sometimes justifiable) and beliefs in mental health stigma relate to the perception of psychological (as opposed to physical) military injuries via both public recognition (such as military honors) and personal support (such as donations to veteran organizations). The authors reported positive associations between masculine honor beliefs and mental health stigma [49].

##### Urbanity and agrarian values

Another approach to studying values in the context of mental health stigma involves the omission of spatial and attitudinal concepts within sources, addressing the relevance of intertwined structural and personal characteristics. escriptions of values being *agrarian* and *suburban* were subsumed within the clusters of milieu specificities as such, e.g., by assessing the role of stoicism in rural Australian residencies [20]. The authors reported both lower scores of stoicism, e.g., lack of emotional involvement, dislike for free emotional expression and the ability to endure emotions, which the researchers introduced as purporting *agrarian* values, and higher levels of self-reported psychological distress independently associated with a greater likelihood of lifetime help-seeking. Furthermore, lower levels of self-efficacy were associated with seeking professional help. However, neither perceived stigma nor attitudes towards seeking professional help explained actual help-seeking in individuals’ history. These results need to be understood within the local organization of healthcare in small rural towns, which are mostly run by general practitioners instead of specialists for psychological problems, thus potentially associated with lower stigma cues. Other researchers focused on *suburban* values, operationalized by factor analysis as a composite variable of “less dense and more politically conservative” areas [22]. Under examination of detailed indicators by zip codes of neighbourhoods in the New York metropolitan area, community members in areas with more *suburban* values reported fewer recovery-oriented attitudes and more microaggressions. In contrast, residential instability and vocational disadvantage were associated with more positive attitudes towards mental illness and fewer microaggressions. In the same survey, community members with psychiatric disabilities reported differences in levels of perceived stigma: people living in congregate housing reported significantly more microaggressions and less physical community participation than those living in independent scattered-side housing [22]. In another study evaluating adherence to general self-reliance in a rural Appalachian sample, higher self-reliance was significantly inversely associated with help-seeking attitudes, with mediation analyses supporting a direct association [19]. In a transnational study, stigmatizing family attitudes were more common in high-income countries, grouped by low endorsement of collectivist values, fewer than three children and high net income, than in low-/middle-income countries [35]. Labelling a condition as “schizophrenia” was associated with more stigmatizing family attitudes in both high-income and low- and middle-income countries, with a stronger association observed in high-income contexts. This suggests that the impact of diagnostic labelling on family attitudes varies across economic and cultural strata [35]. Spahlholz and colleagues examined individuals’ likelihood of seeking help beyond sociodemographic aspects by incorporating a value orientation scale and political attitudes to build a framework on the basis of different milieu groups. Spahlholz et al. reported that *cosmopolitan intellectuals*, characterized by openness to progressive values, are more inclined to utilize professional help [38]. This was contrasted by the findings of *individualists* and *conservatives*, who held more traditional or self-reliant values and reported less readiness to seek help from psychotherapists. However, the likelihood of consulting general practitioners did not differ significantly between groups, highlighting the importance of understanding the complex interplay of values and contextual aspects to improve the utilization of mental health services.

## Discussion

**Fig. 3:**
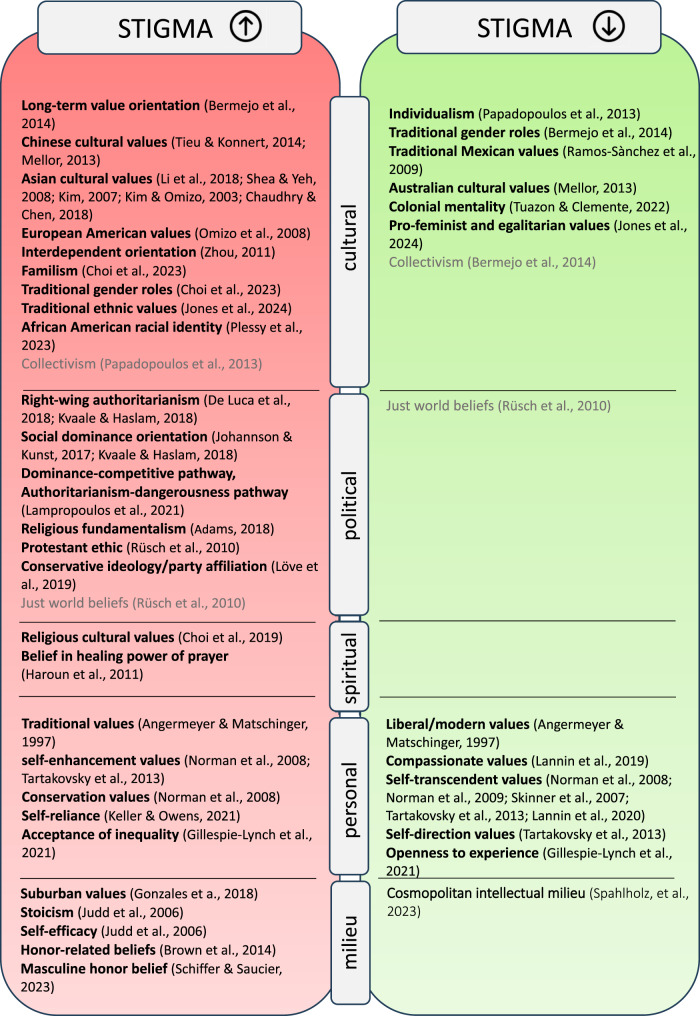
Trends of associations studied between value dimensions and mental health stigma. *Note.* articles with indefinite or conflicting results are coloured in grey letters

The results of this scoping review must be interpreted as preliminary trends and cannot account for causalty. The fragmented evidence and the lack of longituninal and experimental data allowing calls for critical appraisal and empiric reevaluation. Although this review maps a research field at an early stage, we provide an initial summarizing visualization that could attract future research activities as a starting point. A visual overview of associations between values and stigma as studied within the included sources is displayed in Fig. 3. To the best of our knowledge, the present review is the first to compile an overview of the potential relevance of value dimensions for stigma toward people with mental health conditions. This review was conducted on the basis of quantitative evidence with respect to the need to foster a deeper understanding of how specific value dimensions might fuel stigma, which influences the pathways of people living through crises in many detrimental ways. We therefore reviewed sources of evidence on potential links of value dimensions and belief systems with mental health under consideration of cultural and milieu-specific contexts. However, we came across a vastly heterogeneous field in conceptualization and methodology. We were able to include *N* = 49 unique publications that empirically investigated value dimensions in correspondence with reported desires for social distance, emotional reactions, attitudes, help-seeking intentions, and mental health service utilization as dimensions of stigma. By employing a stepwise methodology established for scoping reviews [13], this study provides a comprehensive mapping of the field of study and reports which specific value dimensions are studied in the context of mental health stigma. Stigma towards people living with mental illness generally varies by type of disorder, i.e., alcohol use disorder is rejected the most, followed by schizophrenia, narcissistic personality disorder, depression and panic disorder with agoraphobia [21], in line with the literature on illness-specific aspects of stigma [86]. By condensing the results across all the studies examined, spatial differences and culturally informed aspects were revealed as prominent research foci, e.g., spiritual beliefs about mental health, the role of family members and culture-comparative sampling. Moreover, concepts of political attitudes that are closely connected to values have been studied in different forms by assessing support for authoritarian and conservative views. Another central finding of this work is the lack of stringency and consistency within operationalizations. Despite the increasing number of publications in this area, the vast heterogeneity within and across value dimensions assessed in association with stigma stood out. This contribution was therefore enriched by an overview of the measures used in the study of value domains, allowing a more articulated methodological focus in future research. By synthesizing this heterogeneous evidence, this review might offer starting points for more targeted future research on potential causal interactions and intervention development. The synthesis of evidence further indicates that context specificities fuel or buffer stigma in various forms, e.g., emotional and attitudinal reactions, the willingness to interact, the readiness to discriminate, and to provide different types of support. As Link and Phelan have shown that racism is a fundamental cause of health, not only values play into (self-)stigma and help-seeking intentions but also intersecting experiences by (culturally denominated) minorities within predominant cultures can influence structural accessibility and personal intentions to seek professional support [2].

### Values and beliefs shaped by context (macro level)

Contextual aspects such as social structure, geography and cultural history shape stigma and help-seeking at an overarching level. For example, spatial aspects are of interest when studying cultural values. In areas where *suburban values* are employed (lower housing density and greater conservatism), higher levels of perpetrated stigma by community members are reported. Some authors discuss other spatial-structural aspects as potential influences on the relationship between values and stigma: it is argued that two sociocognitive aspects, dominance-competitiveness and authoritarian-dangerous, vary in their relevance due to contextual structures [77]. The authors reported that the *authoritarian-dangerousness pathway* “will be more significant for contexts where integration is still lacking and people with mental disorders are mostly considered and very often treated in terms of deviance” [77]. On an overarching level, influences that go back to agricultural history were found to be relevant for the predominance of values, as they shape not only economic but also social structure [87]. A focus on group needs and goals over the prioritization of individual needs and goals is central in *collectivist* cultures [4]. Strong group cohesion pronounces a greater distinction from an outgroup and is imperative to conserve ingroup standing [87]. *To save face*, the expression of emotions is rather unfavourable and is experienced as a threat to families’ grace [33]. Some authors suggest that adherence to “Asian values” may lead to a lack of help-seeking attitudes to *save face*. In line with this, Shamblamaw and colleagues found that depression is more stigmatized when so-called *Asian values* (i.e., group cohesion) are employed than when leaning towards more so-called *European values*, i.e., self-enhancement, which are more individualistic goals [30]. Differences in symptom recognition, as well as the expression of *difficult* emotions, help-seeking attitudes and behaviours, can be understood only with respect to contextual value systems. In a Muslim sample, the belief that prayers can heal depression was associated with a lower likelihood of accepting a physician’s diagnosis and taking depression medication; this relationship was mediated by the experience of emotional support through family members [37]. Traditional religious beliefs and family support in coping with mental illness do not necessarily indicate rejection of health care services in general but warrant more culture-sensitive service approaches.

Owing to interdependent orientation [47] or *face concern* [63], physical expression of emotional burden might be more accepted than seeking support for mental health symptoms. This is emphasized by findings indicating that the pronunciation of in-group and family support can be reverse predictors of perceived mental health needs [47]. With the tendency in Asian societies to seek out for general practitioners or alternative healers when mental health issues emerge [75], we know that in the Eastern cultural sphere, more severe symptoms are already displayed when mental health care is sought [88]. Furthermore, culturally informed variation in symptom expression could affect diagnostic accuracy and consequently lead to a diminished fit of treatment within mental health care [89]. This becomes visual with decreased lifetime psychiatric diagnoses for both Latinx and Asian adults, where group-oriented attitudes such as a greater sense of pride and belonging to one´s ethnic group are highly prevalent [90]. Focusing on group needs over individual needs therefore plausibly adds to the understanding of stigma processes at the cultural level. According to Lai et al., Confucianism views mental illness in terms of the sufferer’s dependency on others and their inability to fulfil valued roles and duties [91]. It is perceived to result from a weak character and moral lapse and is viewed as punishment for one’s misdemeanor [92]. As a result, mental illness leads to unstable relationships and marginalization due to “loss of face” within the community, not only for the individual but also for the extended family [92]. Research related to the experiences of stigma among Chinese immigrants to America and Australia diagnosed with mental illness is consistent with this argument [91, 93]. However, individual characteristics such as greater employment of social norms, social dominance orientation, sensitivity to shame brought to the family by depression, and conservative affiliation seem to pronounce such culture-specific findings and underline individual aspects [30]. A systematic review by Villalonga-Olives and Kawachi provides a further perspective from research on social capital informs the complex interplay between social contexts and individual attitudes [94]. The authors of this work highlight that social capital – broadly refering to resources embedded in social networks and mutual obligations- can have both positive and negative effects on health outcomes, depending on the nature and structure of local social ties and contexutal norms. Although not specific to mental health stigma, this contribution underscores that norms and social structures are shaped by underlying value orientations, can shape how individuals perceive, interpret and respond to health-related issues. Such structural consideration highlights how values exist within socio-relational contexts and can influence stigma-dynamics and might affect indiviudal help-seeking behaviour. These contextual influences shape the overarching conditions within which individual value orientations operate. To further understand the potential interplay with mental health stigma, the role of individual value orientations is discussed.

### Individual value orientations and beliefs (micro level)

Values related to a higher degree of stigmatization have been referred to with an indefinite terminology of similar concepts that can be subsumed “self-interested, power-distant, traditionalist, conservative, uncertainty-avoidant, hierarchical, patriarchal value structures”. The participants who leaned to more *traditional values* (achievement, duty/acceptance, materialism) expressed greater desire for distance from individuals with mental health conditions, as a survey conducted in the late 1990s illustrated vicariously [21]. Other research has shown that a preference for power (over universalism) is associated with spontaneous negativity toward ethnic minority groups in implicit tests [95]. This emphasizes the relevance of values associated with self-enhancement attitudes as such potentially fuel discriminatory behaviour. More specifically, respondents who employed rather self-interest, traditionalist, conservative values (as opposed to self-transcendent, compassionate, empathic) were overall more likely to express a desire for social distance and were less prosocial towards people with mental illness in their responses. Furthermore, general self-reliance was linked negatively to attitudes seeking professional help directly and indirectly via greater self-stigma, public stigma and mental health self-reliance [19]. This could be read in line with aspects of perpetuating power and manifesting hierarchy according to the three functions of stigma (keeping people “down, in or away”) [1] and motivational goals of power: To achieve social status and prestige, gaining and controlling dominance over people and resources may be inconsistent with social integration and community living [3]. This is also reflected in political value dimensions and associated attitudes: conservative political self-identification is related to less acceptance of people with mental illness, yet the employment of authoritarian political values predicts stigma more definitively than self-reported political affiliation. However, whereas the authoritarianism measures did not significantly relate to any measure of prejudice in another source [27], preferences for social dominance and just-world beliefs were found to potentially have explanatory value for stigma expression: Employing beliefs in a just world, i.e., assuming that “one gets what they deserve”, was associated with less empathy towards a male vignette displaying symptoms of mental illness.

The value dimensions of tradition, power, and hierarchy are linked to a lower acceptance of people with mental illness. Furthermore, greater acceptance of inequality, less openness, and lower cognitive empathy co-occurred with heightened stigma towards most conditions. The notion of exclusivity in religious fundamentalism, i.e., the rejection of spiritual concepts apart from one’s own affiliation, arguably fits this notion, as it predicted higher rates of stigma independent of Christian orthodoxy. In contrast, Christian Orthodoxy was not predictive of stigma towards people with schizophrenia [36]. The employment of such value dimensions indicates readiness for greater stigma, as they reinforce rigid social structures, reduce openness to different perspectives, and legitimize social inequality. Following Schwartz´s motivational goals of benevolence (preserving and enhancing the welfare of people nearby) and universalism (appreciation of equal rights for all), people who employ higher preferences for self-transcendence values (universalism, benevolence) tend to experience satisfaction and pleasure in caring for others who belong to their own social group [3]. Among the sources included in the present analysis, indeed egalitarian orientation, modern, compassionate, self-transcendence, benevolence, universalism, and openness values are related mostly to less stigma and more positive help-seeking attitudes. For example, leaning towards endorsement of more egalitarian value systems often described within self-transcendence values or “liberal” values (including equality, justice, tolerance) was negatively associated with stigma, as shown by lower levels of desire for social distance [21]. Respondents who employed more compassionate *self-transcendent* values (universalism and benevolence) than *self-interest* values (power and achievement) reported less stigma and more positive help-seeking attitudes [28]. This is reflected in value constructs that touch similar aspects but were studied under different labels. With regard to stigmatizing behavior, one might easily be tempted to put value orientations into boxes of “good” and “bad”. However, we would like to encourage further critical discussion: (a) People with less conservative value orientations might still show stigmatizing behavior since self-reported value orientations could be influenced by socially desireable responses, and (b) the link of more conservative orientations to rejecting people with mental illness could also reflect the differing importance of moral values, i.e., people with a stronger conservative orientation may not have bad intentions in their minds but rather place more value on social order than on individual well-being.

### Limitations

The conflict of highlighting value differences that describe distinctive phenomena at the meso level (milieu specificities) or macro level (cultural context) to understand stigma outcomes at the micro level (daily social interactions) on the one hand and the risk of perpetuating stereotypes and intergroup differences by labelling value clusters on the other hand needs critical reflection. A condensation of different value concepts is needed to bring together fields of research and unify terminology. Thus, a recently proposed condensed framework of cultural elements seems promising [6]. This framework may provide guidance for understanding conceptual similarities and reviewing their applicability rather than adding new terminology to already developed concepts. Such conceptual indifference warrants re-evaluation and distinction of concepts, language and the general framing of mental health/illness. This can arguably be explained by the vast range of value-related aspects potentially influencing stigma, such as different forms of support and behavioural expression within culture and society, as well as health care accessibility and informal care. As all authors of the contribution at hand are of European background, the work is limited by a Western view of the field of research. The search terms used for the present review included only English and German terms and therefore potentially omitted studies that did not provide a title or abstract in such languages. Only one study included data from broader geographical spheres and studied relevant populations under consideration of economic development (e.g., India and Ghana) [35]. Findings need to be understood with respect to “blind spots” of potentially very different forms of rejection, acceptance and support that have not been displayed despite best intentions to do so. Thus, the results of this scoping review lack geographical depth. Although cultural and geographical comparisons were central to the study objectives, the included sources were predominantly sampled within the US, Asia and Europe. This led to a concentration of results emanating from Western societies. Therefore, mental health literacy and the use of services, as determined by Western societies’ understanding of mental health, could have been captured instead of shedding light on stigma mechanisms in different cultures. Further, religious context informed by culture and location such as explanatory models rooted in Buddhist or Hindo traditions, including karmic or reincarnation-based interpretations of illness, may similarly influence stigma towards mental health conditions, however have remained underrepresented in the evidence base at hand. Additionally, prevalent individual values are likely to be an expression of contextual readiness for social change. Understanding forms of stigma expression and experience requires an understanding of its entanglement within more structural components and within time [96]. The view of mental health and normative responsibility for health care might be formed by policies that favour resource distribution for some conditions over others, e.g., structural barriers for people with substance use disorders. Therefore, when cultural differences in stigma and help-seeking are compared, structural differences in the appraisal of mental health in society must be acknowledged as structural influences [77]. A potential limitation of our approach is that the explicit term “help-seeking” was not included in the Boolean operators within the search string. Although we assumed that relevant papers would also contain stigma-related terminology and were therefore unlikely to be missed, we acknowledge that some studies focusing exclusively on help-seeking might not have been identified. Moreover, the findings of this review are shaped by abductive definition of the clusters presented. This approach may have biased both the interpretation and the organization of the extracted information. At the same time, it served as a pragmatic strategy to reduce complexity within a highly heterogeneous research field and provided a first step towards systematically mapping the role of values dimensions in the context of mental health stigma. As a growing body of research indicates that personal contact of professional familiarity with people with mental illness can shape the relationship between values and stigma in direct and indirect ways [97], future research warrants close consideration of the potential intermediate role of exposure to mental health conditio. Another study suggests, that personal experience with metal illness does not uniformly translate into reduced stigma, highlighting the complexity of interrelated individual and contextual aspects at play [98]. In the present study, contact hypotheses were not exclicitly adressed as potential intermediate factors, which might limit the conceptual depth. Further examination is needed to understand the potentially complex interplay on how contact may shape mechanisms linking value orientations to stigma-related attitudes. A formal quality assessment allowed for enhanced interpretability of reported findings. Whilst most included sources of evidence compile sound methodological frameworks over all, shortcomings of quality on study level were predominantly lacking justifications for sample size as well as sampling procedures. The scoping review has been limited to quantitative research, thus excluding the potential of more in-depth approaches, e.g., content analysis, that could provide extended insight via subjective expressions and experiences in qualitative data.

### Conclusion and implications

The substantial heterogeneity of value-related aspects studied in the field of mental health stigma stood out. Although it has been evaluated from different perspectives within a growing body of research in the field of human values and mental health, a granular focus and a high degree of variability remain prevalent, with discordant consensus. This can be explained in part by the indefinite wording of concepts and overlapping frameworks [6]. Differences in the conceptual provenance as well as the characteristics of the samples under study therefore do not allow for the generalization of the results at the current stage. Stigma underlies complex interpersonal processes that are formed and perpetuated by social hierarchy, prominently referred to as *stigma power* [2]. This review highlights the relevance of *what matters most* to individuals and highlights first the tendencies of how such values might motivate stigma at the individual and societal levels, i.e., policies, laws, and decisions related to resourced distribution. The present review underlines the importance of different values and belief systems regarding stigma towards mental health conditions in light of cultural imprints of how mental health conditions are perceived, described and addressed. The synthesis of evidence further indicates that context specificities fuel or buffer stigma in various forms, e.g., emotional and attitudinal reactions, the willingness to interact, the readiness to discriminate, and to provide different types of support. Thus, future research may benefit from study designs that more directly examine mechanisms through which stigma can be reduced. Longitudinal and interventional approaches could help to clarify how value orientation and belief systems influence different levels of stigma over time and whether there factors can be modified by targeted stigma-reduction strategies, e.g. acknowledging own experience of mental health crises of contact to people living with mental health problems. As Link and Phelan have shown that racism is a *fundamental cause* of health, not only values play into (self-)stigma and help-seeking intentions but also intersecting experiences by (culturally denominated) minorities within predominant cultures can influence structural accessibility and personal intentions to seek professional support [2]. Considering social functions of stigma as a reaction to what is most at stake, future research interested in the link between values and stigma should also address more distal circumstances (e.g., the distribution of power and wealth, accessibility to social and health-care-related resources, and policies) that similarly affect stigma at the structural level. In a methodological approach, Hanel and colleagues emphasize that the illustration and interpretation of quantitative data in attitude research must be complemented by the denomination of observed similarities [99]. This should be accounted for in future work on values and mental health stigma, since, as Jo Cox (2015) expressed in her maiden speech, “*We have far more in common with each other than things that divide us.”* [100].

### Future directions for research angendas

1. Conceptual clarification and unified use of terminology within values studied in the field of mental health stigma are warranted.
2. Re-evaluations of values dimensions within various cultural spaces, including sources beyond English and German-speaking publications, are needed.
3. The development of validated operationalization tools for assessing values and stigma in a context-sensitive way to enable more consistent and comprehensive research (e.g., the value-based stigma inventory (VASI)) [10].
4. Longitudinal studies are warranted to explore causal relationships and manifestations between value dimensions and stigma while considering the development of interpersonal motives at the macro (culture, country) and meso levels (family, peers, milieu) over time [101].
5. Examination of the interplay between individually employed values, sociocultural context, own experiences or contact to people with mental health problems, prevalent etiology beliefs and real-life structural barriers to mental health equity, including the experiences and expertise of those adressed by mental health services.

Detailed information of the final selection of articles is condensed for each source and can be found in Table 3 below.

**Table 3:** Main characteristics and results of the included sources

Description of Articles				Sample		Value Dimension	Methods			
Authors	aims	findings		Sample, *N*, (m%/f%/o%), age (range)	country		Group comparison	Vignettes	Surveys	F-2-f
							12	16	46	3
Angermeyer & Matschinger (1997)	social distance towards mentally ill (schizophrenia, depression, alcohol dependence, panic disorder, agoraphobia, narcistic personality disorder)	The study found that respondents with traditional values (e.g., duty, materialism) preferred greater distance from individuals with mental illnesses. In contrast, those endorsing liberal (equality, tolerance) or modern values (self-realization, hedonism) showed greater acceptance.	traditional values ▲ public stigma liberal/modern values ▼public stigma	general population, *N* = 1022–3114	GER	personal, milieu-specific	no	x	x	x
Kim & Omizo (2003)	examine the relationships among Asian American adherence to Asian cultural values, attitudes toward seeking professional psychological help, and willingness to see a counsellor	The study found that adherence to Asian cultural values was inversely associated with both attitudes toward seeking professional psychological help and the willingness to see a counsellor, even after accounting for demographic factors. Additionally, attitudes toward seeking professional psychological help fully mediated the relationship between adherence to Asian cultural values and the willingness to see a counsellor, both for general purposes and for addressing personal and health-related issues.	Asian values ▼ help-seeking attitudes mediating effect of attitudes toward seeking professional psychological help on the relationship between adherence to Asian cultural values and willingness to see a counsellor in general	university/ college students, *N* = 242 (42.1%/57.9%/0%) *M* = 22.0 years (18–57 years)	US	cultural	no		x	
Beck, Angermeyer & Brähler (2005)	investigation of association between right-wing extremist orientation and attitude regarding people with mental illness	The study found a marginal and inconsistent correlation between right-wing extremism and social distance towards mentally ill individuals, with only 5% of the variance explained.	right wing extremism ⬧ social distance towards the mentally ill	general population, *N* = 2089 (>14 years)	GER	political	no		x	
Judd et al. (2006)	examine the role of stoicism, self-efficacy, and perceived stigma in predicting help-seeking by rural residents, for mental health problems	Lower scores in both stoicism and general self-efficacy were associated with a higher likelihood of having sought professional help.	stoicism ▼ help-seeking in history self-efficacy ▼ help-seeking in history	general population, *N* = 467 (41.2/58.8%/0%) *M* = 56.6 years	AUS	personal, milieu-specific	no		x	
Skinner et al. (2007)	examining the role of values, affect, and deservingness judgments in health professionals’ views of patients with stigmatized conditions	The study found that general value orientations significantly influenced affective reactions toward drug users. Conservation values (e.g., conformity, security, tradition) were linked to increased negative affect (e.g., anger, disappointment) and higher perceived responsibility for heroin use. In contrast, self-transcendence values (e.g., empathy, care for others) were associated with stronger positive affect (e.g., sympathy, concern) toward drug users. Importantly, conservation values did not reduce positive affect, nor did self-transcendence values diminish negative affect in the path analysis.	conservation values ▲ public stigma self-transcendence values ▼ public stigma	practitioner/ professionals/ nurses, *N* = 277 *M* = 48.1 years (22 - 75 years)	AUS	personal	no	x	x	
Kim (2007)	possible relations among enculturation and acculturation to cultural values and attitudes toward seeking professional psychological help were examined; additionally: possible relations between various dimensions of Asian values and attitudes toward seeking professional psychological help were examined.	The results demonstrated a significant inverse relationship between enculturation to Asian cultural values and attitudes toward seeking professional psychological help. This relationship persisted even when controlling for acculturation to European American values and the influence of previous counselling experience.	Asian cultural values ▼ help-seeking attitudes	university/ college students, *N* = 146 (33.6%/66.4%/0%) *M* = 20.0 years (18–36 years)	US	cultural	no		x	
Norman et al. (2008)	investigating the relation of responses to the Schwartz Value Scale to preferred social distance to a person with either schizophrenia or depression	Three of the value orientations show significant correlations with social distance: Greater self-transcendence was associated with greater willingness to interact with the person with mental illness while self-enhancement or conservatism value orientation was associated with preference for greater social distance.	Greater self-transcendence ▼ public stigma self-enhancement or conservatism ▲ public stigma	university/ college students, *N* = 200 (45.0%/55.0%/0%) *M* = 21.5 years	CAN	personal	no	x	x	
Omizo et al. (2008)	examining the extent to which Asian American adolescents who were living in Hawaii adhered to Asian and European American cultural values in relation to mental health variables	Adherence to European American values was negatively associated with a positive attitude toward seeking professional psychological help.	European American values ▼ help-seeking attitudes	high school students, *N* = 112 (41.0%/58.0%/1.0%) *M* = 16.8 years (15–19 years)	US	cultural	no		x	
Shea & Yeh (2008)	exploring how adherence to Asian values, stigma associated with receiving psychological help, relational-interdependent self-construal, age, and gender individually and collectively predicted attitudes toward seeking professional psychological help among Asian American college and graduate students; examining how stigma mediates the relationship between adherence to Asian values and help-seeking attitudes	Lower adherence to Asian values, lower levels of stigma, a higher relational-interdependent self-construal were associated with more positive help-seeking attitudes. The mediation model was not significant.	Asian cultural values▼ help-seeking attitudes	university/ college students, *N* = 219 (35.0%/65.0%/0%) *M* = 24.6 years (18–35 years)	US	cultural	no		x	
Norman et al. (2009)	Self-transcendent value priorities and attitudes toward a young woman described as having schizophrenia were assessed	Those who provided a higher endorsement of self-transcendent values also indicated a more positive attitude toward the ill person, but neither attitude to the person nor self-transcendent values showed a zero-order correlation with seating distance. However, the interaction between these values and attitudes significantly influenced physical proximity: When self-transcendent values were high, attitudes did not significantly predict seating distance. In contrast, when self-transcendent values were low, more positive attitudes were associated with closer seating distance.	self-transcendent values ▼ public stigma self-transcendent values ⬧seating distance moderating effect of self-transcendent values on the relationship between attitudes toward people with a mental illness and seating distance	university/ college students, *N* = 95 (36.8%/63.2%/0%) *M* = 18.8 years	CAN	personal	no	x	x	
Ramos-Sánchez et al. (2009)	investigation of the relationships between Mexican acculturation, cultural values, gender, and help-seeking intentions among Mexican American community college students	More enculturated Mexican Americans reported greater positive attitudes toward help seeking than did less enculturated Mexican Americans.	traditional Mexican culture and values ▲ help-seeking attitudes	university/ college students, *N* = 262 (31.0%/69.0%/0%) *M* = 27.0 years (16–60 years)	US	cultural, religious	no		x	
Rüsch et al. (2010)	examining the link between meritocratic worldviews and mental illness stigma, among both members of the public and people with mental illness	The study found a consistent positive association between endorsing the Protestant ethic and stigmatizing self-reported attitudes in both the general public and individuals with mental illness. Among the public, implicit guilt-related stereotypes were also linked to the Protestant ethic. For individuals with mental illness, stronger just world beliefs about oneself were associated with reduced self-stigma but increased implicit self-blame.	Protestant ethic ▲ self-stigma Only public: Protestant ethic ▲ Implicit ‘Mental Illness–Guilty’ Association Only individuals with mental illness: just world beliefs ▼ self-stigma just world beliefs ▲ Implicit ‘Mental Illness–Guilty’ association	general population, people with mental illness, *N* = 135 mental illness sample: *n* = 85 (68.0%/32.0%/0%) *M* = 44.8 years control sample: *n* = 50 (70.0%/30.0%/0%) *M* = 45.0 years	US	political	no		x	
Haroun et al. (2011)	investigating barriers and suggest treatment models for depressive disorders in Muslim adolescents and young adults residing in the United States	Most responders believed that recitation from the Koran relieves mental distress. Those who believed prayer could heal depression were less likely to accept a physician’s diagnosis, even when controlling for age, gender, and ethnicity.	believing prayer can heal depression ▼ diagnosis acceptance	young adults, *N* = 125 (60.0%/40.0%/0%) *M* = 17.7 years (14–21 years)	US	religious	no		x	
Papadopoulos et al. (2013)	investigating whether the cross-cultural value paradigm ‘individualism-collectivism’ is a useful explanatory model for mental illness stigma on a cultural level	Higher scores of individualism in cultural groups correlated with less stigmatizing attitudes, whereas higher scores of collectivism correlated with more stigmatizing attitudes.	individualism ▼ public stigma collectivism ▲ public stigma	general population, *N* = 305 (47.2%/52.8%/0%) *M* = 30.0 years (18–82 years)	UK/England	cultural	yes		x	
Tartakovsky et al. (2013)	connections between the value preferences, attitudes toward community living, and burnout among staff members of community services for people with intellectual disability (*n* = 126) and severe mental illness (*n* = 96) in Israel	High preference for the self-transcendence values (both benevolence and universalism) and a low preference for the self-enhancement value of power were associated with the staff members’ positive attitude toward their clients’ empowerment, a higher sense of similarity, and a negative attitude toward exclusion. In addition, a high preference for the values of self-direction, which belongs to the openness to change value type, was associated with a positive attitude toward empowerment, a high sense of similarity, and a negative attitude toward exclusion.	self-transcendence values ▼ public stigma self-enhancement values ▲ public stigma self-direction values ▼ public stigma	practitioner/ professionals/ nurses, *N* = 222 (23.0%/77.0%/0%) *M* = 36.2 years	Israel	personal	no		x	
Mellor (2013)	investigating the differences in levels of stigmatizing attitudes toward people with mental illness between Chinese individuals in their home country (Taiwanese), Chinese immigrants to Australia, Australian-born Chinese, and Anglo-Australians; assessing whether level of acculturation (rather than length of stay in the new country) plays a role in stigma	Chinese immigrants and Taiwanese held significantly more stigmatizing attitudes toward people with mental illness than Australian-born Chinese and Anglo-Australians. Endorsement of mainstream (Aus) cultural practices by Australian-born Chinese and Chinese immigrants was associated with significant lower levels of stigmatizing attitudes related to competence, dangerousness, and social distance in both groups. Endorsement of heritage (Chinese) cultural practices was associated with higher attributions of dangerousness to the people with mental illness within both groups.	Australian cultural values ▼ public stigma Chinese cultural values ▲ public stigma	general population, *N* = 543 (36.1%/63.9%/0%) *M* = 38.0 years (19–92 years)	Taiwan, AUS	cultural	yes		x	
Bermejo et al. (2014)	Analysis of the influence of cultural factors on the attitude towards use of psychological-psychiatric measures	A collectivist mindset, and traditional gender roles were linked to more positive attitudes toward using psychological services. Meanwhile, long-term value orientation and low psychopathological stress were associated with a more hesitant approach to seeking help.	collectivism ▲ help-seeking attitudes traditional gender roles ▲ help-seeking attitudes long-term value orientation ▼ help-seeking attitudes	general population, *N* = 626 (30.8%/69.2%/0%) *M* = 32.3 years	GER	cultural	yes		x	
Brown et al. (2014)	Study 1 uses an individual level of analysis, connecting the endorsement of honour-related beliefs and values to attitudes toward seeking mental healthcare.	Individuals who strongly endorsed honor-related beliefs and values were especially concerned that seeking help for mental health needs would indicate personal weakness and would harm their reputations. Honor states in the U.S. invested less in mental health care resources and parents living in honor-states were less likely to use mental health services on behalf of their children.	honor-related beliefs ▲ self-stigma honor-related beliefs ▲ perceived stigma	university/ college students, *N* = 756 (31.1%/65.9%/0%) *M* = 19.0 years	US	–milieu- specific	no		x	
Tieu & Konnert (2014)	determining the extent to which demographic factors, perceived social support, and Chinese cultural beliefs predict attitudes toward mental health help seeking; assessing mental health utilization; assessing intentions to utilize mental health services among older Chinese immigrants in Canada aged 55 and above	The study found that demographic and health information, perceived social support, and Chinese cultural beliefs and values explained 21.8% of the variance in help-seeking attitudes. However, these older participants exhibited significantly less positive help-seeking attitudes compared to a community sample in Canada, with their attitudes strongly linked to Chinese cultural beliefs and values.	Chinese cultural beliefs and values ▼ help-seeking attitudes	“Older age”, *N* = 157 (30.0%/70.0%/0%) *M* = 73.9 years (55–92 years)	CAN	cultural	no		x	x
Shamblaw et al. (2015)	examining differences in depression stigma among Asians and Europeans and the mediating mechanisms	The study indicated that individuals of Asian origin stigmatize individuals with depression more than do individuals of European origin. The belief that depression brings shame to one’s family, perception of social norms, a social dominance orientation, and conservative values were significant mediators between ethnicity and depression stigma (social distance and depression attribution) with perceived norms and familial shame having the largest indirect effects.	relationship between ethnicity and depression stigma mediated by effect of social norms, the belief that depression brings shame to one’s family, a social dominance orientation, and conservative values	university/ college students, *N* = 478 (38.0%/62.0%/0%) European *n* = 199 (27.6%/72.4%/0%) *M* = 18.3 years (16–26 years) Asian *n* = 249 (46.2%/53.8%/0%) *M* = 19.0 years (16–41 years)	CAN	personal	yes	x	x	
Wesselmann, 2015	investigating how religious beliefs about mental illness influenced the types of social support individual would be willing to give persons with mental illness	This study identified three types of social support: “spiritual social support,” “secular counselling support,” and “secular instrumental support.” Evangelical Protestant Christians were more likely than Roman Catholic Christians to prefer providing spiritual social support, while no significant group differences were found for the secular support types. Religious beliefs influenced support preferences, with Christians who viewed mental illness as stemming from immorality, sinfulness, or spiritual causes being more likely to prefer spiritual social support for a hypothetical friend with depression. Evangelical Christians were more likely than Mainline Protestant and Roman Catholic Christians to endorse spiritual explanations for mental illness and to prefer spiritual social support.	Evangelical Protestant Christians were more likely than Roman Catholic Christians to prefer providing spiritual social support. Evangelical Christians were more likely than Mainline Protestant and Roman Catholic Christians to endorse spiritual explanations for mental illness and to prefer spiritual social support	university/ college students, *N* = 164 *M* = 19.2 years	US	religious	yes	x		
Kvaale & Haslam (2016)	shedding light on the motives that underpin stigmatizing attitudes, and investigating if these motives also predict how people interpret biogenetic explanations	Right Wing Authoritarianism (RWA) and Social Dominance Orientation (SDO) were independently associated with higher levels of each facet of stigmatizing attitudes toward persons with depression and schizophrenia. Additionally, the motives indexed by SDO and RWA predicted how people responded to a biogenetic explanation of schizophrenia, tending to reinforce stigmatizing attitudes.	Right Wing Authoritarianism ▲ public stigma Social Dominance Orientation ▲ public stigma	university/ college students, *N* = 177 (23.2%/76.8%/0%) *M* = 19.6 years (17–47 years)	AUS	political	no	x		
Johannson & Kunst (2017)	comparing the explanatory power of individual differences in three socio-political attitudes (Social Dominance Orientation, Right-Wing Authoritarianism, and Belief in a Just World) and of characteristics of the mentally ill such as their social status, gender, and type of disease; testing whether both types of variables interactively predict prejudice	Social Dominance Orientation predicted higher levels of prejudice in both a general population sample and a sample of health professionals and trainees, although the effect was less pronounced among health professionals. Right-Wing Authoritarianism and Belief in a Just World did not significantly predict prejudice. Belief in a Just World interacted with the target’s gender, leading to less empathy toward a male compared to a female mentally ill person.	Social Dominance Orientation ▲ public stigma Right-Wing Authoritarianism ⬧ public stigma Belief in a Just World ⬧ public stigma	general population, *N* = 169 (37.9%/62.1%/0%) *M* = 26.2 years	NOR	political	no	x	x	
Li et al., 2017	investigating help-seeking intentions and use of mental health services based on a model of Chinese students’ help-seeking intent	The results suggested that social-cognitive variables had significant effects both on students’ intentions to seek professional mental health care and their actual service use. The hypothesized model of help-seeking intentions exhibited a good fit to the data. Subjective norms were found to be a significant mediator of the relationship from evaluated need and anticipated risks to help-seeking intentions. The current findings indicated that neither adherence to Asian values nor social support significantly impacted students ‘help-seeking activities.	Asian cultural values ⬧ help-seeking in history mediating effect of subjective norms on the relationship from evaluated need and anticipated risks to help-seeking intentions	university/ college students, *N* = 1128 (55.9%/44.1%/0%) *M* = 20.0 years (17–45 years)	CHI	cultural, milieu-specific	no		x	
Gonzales et al. (2018)	examining the association between neighbourhood characteristics, stigma related to mental illness reported by local community members, and measures of perceived stigma and community participation among individuals with psychiatric disabilities living in independent scattered-site housing or in congregate housing in three neighbourhoods in the NYC metropolitan area.	Community members in areas with more “suburban values” (less dense and more politically conservative) also reported fewer recovery-oriented attitudes (AMIS recovery) and more microaggressions.	suburban values ▲ public stigma suburban values ▲ microaggressions	general population, people with mental illness, *N* = 951 Community members*: n* = 608 (59.0%/41.0%/0%) *M* = 42.0 years Participants with psychiatric disability: *n* = 343 (62.0%/38.0%/0%) *M* = 47.9 years	US	milieu-specific	yes		x	x
Chaudhry & Chen (2018)	investigation first compared endorsement of stigmatizing beliefs and attitudes toward mental illness (onset responsibility and courtesy stigma) among South Asian Americans, East Asian Americans, and European Americans, then tested associations between cultural mechanisms, education in psychology, and endorsement of mental illness stigma	South Asian Americans exhibited greater endorsement of courtesy stigma compared to European Americans, while perceptions of onset responsibility did not differ across the three ethnic-cultural groups. Endorsement of Asian cultural values were positively associated with courtesy stigma and endorsement of onset responsibility; however, these associations were only significant in the full sample and among European Americans. Higher adherence to traditional Asian values was linked to increased courtesy stigma and onset responsibility, but these associations were moderated by ethnicity and were significant only for European Americans.	Asian cultural values ▲ public stigma moderating effect of ethnicity on the relationship between Asian values and courtesy stigma/onset responsibility	university/ college students, *N* = 189 (48.0%/52.0%/0%) *M* = 20.4 years (18–33 years)	US	cultural	yes	x	x	
Li et al. (2018)	investigating correlates of Australian university students’ help-seeking intentions and actual service usage	The endorsement of Asian values associated negatively with intentions and usage of help-seeking. Relationships between several predictors (knowledge concerning mental health and services, evaluated and perceived need, anticipated benefits, stigma concerns, and Asian values) and help-seeking intentions were significantly mediated by attitudes toward help seeking and subjective norms	Asian cultural values ▼ help-seeking in history Asian cultural values ▼ help-seeking attitudes mediating effect of attitudes towards help-seeking and subjective norms on the relationship between several predictors (knowledge concerning mental health and services, evaluated and perceived need, anticipated benefits, stigma concerns, and Asian values) and help-seeking intentions	university/ college students, *N* = 611 (34.0%/66.0%/0%) *M* = 21.0 years	AUS	cultural	no		x	
De Luca et al. (2018)	investigating the association between right-wing authoritarianism and multiple stigma outcomes, including stereotypes, attributions/negative affect, social distance, microaggressions and help-seeking self-stigma	Findings indicated that right-wing authoritarianism was significantly associated with all mental health stigma dimensions analysed, even after controlling for covariates	right-wing authoritarianism ▲ self-stigma right-wing authoritarianism ▲ perceived stigma	general population, *N* = 518 (49.2%/50.6%/0%) *M* = 46.4 years (18–82 years)	US	political	no	x	x	
Adams, 2018	examining the extent to which Christian orthodoxy and religious fundamentalism were associated with participant attitudes to- ward individuals with a mental illness (schizophrenia), with a general medical illness (diabetes), and with no illness (with a practicing Christian as the control condition)	The study found a small but significant relationship between religious fundamentalism and negative attitudes toward individuals with mental illness (schizophrenia). Religious fundamentalism, but not Christian orthodoxy, significantly predicted stigmatizing attitudes toward mental illness. Neither religious fundamentalism nor Christian orthodoxy predicted stigma toward a general medical illness (diabetes). However, both were significant predictors of positive attitudes toward a practicing Christian, consistent with previous research on stigmatized groups.	religious fundamentalism ▲ public stigma Christian orthodoxy ⬧ public stigma	university/ college students, *N* = 204 (24.5%/75.5%/0%) *M* = 22.8 years (18–47 years)	US	religious	yes	x		
Lannin et al. (2019)	examination of the association between medical students’ compassionate values, help-seeking stigma, and help-seeking attitudes	Compassionate values were negatively associated with stigma. Stigma, in turn, was negatively associated with help-seeking attitudes. Additionally, there was a statistically significant indirect effect of compassionate values on help-seeking attitudes through stigma. This indicates that prioritizing compassionate values over self-interested values is linked to more positive help-seeking attitudes due to the negative association with stigma.	compassionate values ▼ self-stigma moderating effect of self-stigma on the relationship between compassionate values and help-seeking attitudes	university/ college students, *N* = 91 (41.8%/57.1%/1.1%)	US	personal	no		x	
Choi et al. (2019)	study focuses on direct and indirect effects of religious cultural values (Spiritual etiology beliefs) reported by Mexican American college women and how such can shape their willingness to seek counselling	Religious cultural values indirectly related to a diminished willingness to seek counselling through beliefs in spiritual etiology and self-stigma. However, religious cultural values were not directly linked to self-stigma or willingness to seek counselling. This suggests that religious cultural values may subtly shape cultural understandings, perceptions of mental illness causes, and culturally acceptable coping practices.	religious cultural values ▼ help-seeking attitudes moderating effect of religious cultural values on seeking counselling although beliefs in spiritual etiology and self-stigma	university/ college students, *N* = 276 (0%/100%/0%)	US	religious	no		x	
Löve et al. (2019)	investigating the relationship between political ideology and stigmatizing attitudes toward depression in Sweden	More conservative ideology and party affiliation were associated with higher stigmatizing attitudes toward depression. Conservative party supporters were more likely to believe that people could “snap out of” depression if they wanted to, while populist right-wing supporters more often viewed individuals with depression as “dangerous” and “unpredictable.” Self-stigma was also highest among populist right-wing supporters. These findings support both hypotheses: conservative ideology is linked to greater stigma toward depression, and differences exist among conservative supporters, with traditional conservatives emphasizing personal agency and populist right-wing supporters focusing on unpredictability and fear.	conservative ideology ▲ public stigma conservative party affiliation ▲ public stigma	general population, *N* = 3246 (52.7%/47.3%/0%)	SVE	political	yes		x	
De Vitre (2020)	exploring the relation between Asian Americans acculturation and enculturation as factors mediating and moderation effects of Asia Americans attitudes towards seeking mental health services	Both enculturation and acculturation values served as mediators in the relationship between ethnicity and attitudes toward seeking mental health services, with enculturation showing a full effect and acculturation demonstrating a partial effect. These findings suggest that enculturation significantly and negatively influences attitudes toward seeking mental health services, while acculturation exerts a significant but partial positive influence on these attitudes.	mediating effect of enculturation values and acculturation values on the relationship between ethnicity and attitudes towards seeking mental health services	older age, *N* = 75 (60.0%/40.0%/0%) *M* = 29.0 years (18–76 years)	US	cultural	no		x	
Lannin et al. (2020)	examining how personal values predicted public stigma and self-stigma of seeking psychological help	The findings supported the hypothesis that prioritizing self-transcendence values is associated with lower stigma. Specifically, self-transcendence had a direct negative relationship with self-stigma and an indirect negative relationship with public stigma, suggesting that prioritizing the well-being of others reduces both personal and public stigmatizing beliefs about seeking psychological help. Contrary to predictions, self-enhancement values were not significantly linked to higher self-stigma through public stigma in the final model, despite significant zero-order correlations with both stigma variables. Additionally, openness to change and conservation values did not predict public stigma or have significant indirect effects on self-stigma through public stigma.	self-transcendence values ▼ public stigma and self-stigma	university/ college students, *N* = 342 PWI sample: *n* = 244 (21.0%/79.0%/0%) *M* = 20.3 years (18–54 years) HBCU *n* = 98 (17.0%/83.0%/0%) *M* = 19.7 years (18–26 years)	US	cultural, personal	yes		x	
Chen et al. (2020)	examining cultural influences on stigma toward mental illness and perceived barrier to help-seeking among Hong Kong Chinese, Chinese Americans, and European Americans	Hong Kong Chinese reported the highest stigma and barriers, followed by Chinese Americans, with European Americans reporting the lowest. Both Chinese groups exhibited significantly higher face concern than European Americans, with no significant difference between the two Chinese groups. Face concern significantly mediated cultural differences in stigma and barriers to help-seeking for both Hong Kong Chinese and Chinese Americans compared to European Americans.	mediating effect of face concern on the relationship between cultures and stigma/barriers to help seeking	university/ college students, *N* = 555 Hong Kong Chinese: *n* = 170 (42.9%/57.1%/0%) *M* = 19.6 years Chinese Americans: *n* = 194 (32.0%/68.0%/0%) *M* = 19.3 years European Americans: *n* = 191 (45.6%/54.4%/0%) *M* = 19.9 years	US, CHI	cultural	yes		x	
Zhou (2021)	examine shared relationships between cultural values, mental health attitudes, and mental health behaviours (perceived mental health treatment need and help-seeking) in Asian and Latinx college students	Students from both Asian and Latinx backgrounds shared two cultural values: interdependent orientation (IO), emphasizing the importance of respecting and supporting one’s family and in-group, and cultural obligation (CO), reflecting a sense of duty to maintain and participate in one’s broader culture. Regardless of ethnicity, the more students endorsed IO values, the less likely they were to perceive a need for mental health treatment. IO value adherence was also predictive of more negative attitudes towards mental health. CO values were not predictive of perceived need or help-seeking behaviours.	interdependent orientation ▼ help-seeking attitudes interdependent orientation ▲ public stigma cultural obligation ⬧ perceived needs or help-seeking in history	university/ college students, *N* = 159 (39.0%/61.0%/0%)	US	cultural	no		x	
Keller & Owens (2021)	examination of four - stage chain of serial mediation where higher levels of general self-reliance would be related to greater levels of public stigma which would in turn be related to higher levels of self-stigma, followed by greater self-reliance about managing mental health problems and finally more negative attitudes toward seeking help from psychologist	General self-reliance had a positive direct link to public stigma, self-stigma, and mental health self-reliance, along with a negative direct link to attitudes toward seeking professional psychological help. Association between general self - reliance and attitudes toward help seeking that was explained in serial by higher levels of public stigma, self-stigma, and mental health care. Significant four-stage chain of mediation from general self-reliance to attitudes toward seeking professional psychological help via higher public stigma, self-stigma, and self-reliance about coping with mental health problems in serial.	self-reliance ▲ public stigma, self-stigma and mental health self-reliance self-reliance ▼ help-seeking attitudes	general population, *N* = 783 (12.0%/88.0%/0%) *M* = 52.4 years	US	personal	no		x	
Serpas (2021)	examination of the moderating effect of the intrinsic motivation on everyday life discrimination as a protective factor against reduced mental health among Hispanic/Latinx undergraduates	Findings reveal a crossover interaction, with a buffering effect of intrinsic motivation observed with only depressive symptoms. A significant effect was detected only among participants endorsing high level of discrimination suggesting that intrinsic value motivation was only protective when faced with high levels of discrimination.	moderating effect of relative extrinsic-intrinsic value orientation on the relationship between everyday discrimination and depressive symptoms	university/ college students, *N* = 174 (18.0%/82.0%/0%) *M* = 22.4 years	US	personal	no		x	
Lampropoulos et al. (2021)	applying the Dual-Process Cognitive-Motivational Model of Ideology and Prejudice to the study of stigmatizing attitudes in two contexts where the model has not been tested before: France (*N* = 224) and Greece (*N* = 238); testing the relation of the model’s two pathways (dominance-competitiveness and authoritarianism-dangerousness) to stigmatizing attitudes	The models adequately fit the data and partially supported prior findings. In the Greek sample, however, the dominance-competitiveness pathway was not significantly linked to stigmatizing attitudes, indicating that stigma in this group is more associated with deviance and threat.	French sample: dominance-competitive pathway ▲ public stigma authoritarianism-dangerousness pathway ▲ public stigma Greek sample: dominance-competitive pathway ⬧ public stigma authoritarianism-dangerousness pathway ▲ public stigma	general population, university/ college students, *N* = 462 French: *n* = 224 (24.6%/75.4%/0%) *M* = 36.4 years Greek*: n* = 238 (37.0%/63.0%/0%) *M* = 33.7 years	FRA, GREE	cultural, political	no		x	
Gillespie-Lynch et al. (2021)	comparing stigma towards students with the following conditions among university students in the US and Lebanon: withdrawn and disruptive autism, learning disability (LD), ADHD, social anxiety, depression, mania, eating disorders, addiction to pain medication, psychopathy, and schizophrenia	This study found that perceived dangerousness predicted autism stigma and greater acceptance of inequality, lower openness to experience, and reduced cognitive empathy were associated with heightened stigma toward most mental health conditions. Expanding on prior research on autism, the findings suggest these predictors of stigma are relevant across a broader range of conditions. Diagnostic labels were generally less stigmatized than associated behaviours.	acceptance of inequality ▲ public stigma openness to experience ▼ public stigma cognitive empathy ▼ public stigma	university/ college students, *N* = 907 U.S. American: *n* = 633 (43.6%/56.4%/0%) *M* = 19.8 years Lebanese: *n* = 274 (46.5%/53.5%/0%) *M* = 18.8 years	US, Lebanon	cultural, personal	no	x	x	
Wusten & Lincoln (2022)	determine whether stigmatizing family attitudes are less prevalent in LAMIC than in HIC and whether stigmatizing family attitudes are intensified in both country types by introducing a schizophrenia label	Participants from LAMIC exhibited significantly fewer stigmatizing attitudes toward family members with schizophrenia compared to those from HIC. Introducing a schizophrenia label increased stigmatizing attitudes in both groups, but the effect was stronger in HIC. Post hoc analyses revealed that the country type significantly influenced most family attitude variables, except anger and empathy. Labelling had a significant effect on family attitudes, with more stigma observed in the labelled condition. Furthermore, an interaction effect showed that the impact of labelling on family attitudes was more pronounced in HIC than in LAMIC. These findings highlight differences in stigma and labelling effects based on economic and cultural contexts.	LAMIC ->fewer stigmatizing attitudes compared to HIC	general population, *N* = 1474 HIC: *n* = 711 (48.5%/51.5%/0%) *M* = 33.9 years LAMIC: *n* = 763 (77.9%/22.1%/0%) *M* = 27.8 years	US, Ghana, India, Columbia, Mexico & UK/England	cultural, milieu-specific	yes	x	x	
Tuazon & Clemente (2022)	examining how acculturation and enculturation mediate the relationship between colonial mentality (CM) and mental health help-seeking attitudes, including psychological openness, indifference to stigma, and help-seeking propensity among Filipino American participants	Only help-seeking propensity was significantly predicted by CM, independent of acculturation and enculturation. However, mediation analysis revealed that acculturation mediates the relationship between CM and psychological openness, while interpersonal norms, a component of enculturation, mediate the impact of CM on both help-seeking propensity and indifference to stigma.	colonial mentality ▲ help-seeking attitudes mediating effect of acculturation on the relationship between CM and psychological openness mediating effect of interpersonal norms on the relationship between CM and help-seeking propensity as well as indifference to stigma	general population, *N* = 218 (39.4%/58.3%/2.3%)	US	cultural	no		x	
Choi et al. (2023)	examining the impact of multidimensional cultural values, such as familism, respect, religiosity, traditional gender roles (machismo and marianismo), and subjective social class on mental health and attitudes toward seeking counselling, with generational status as a moderator	The study found that better mental health was associated with lower adherence to the familism value, higher adherence to the respect value, and a higher subjective social class. Additionally, more positive attitudes toward seeking counselling were linked to lower adherence to familism and traditional gender role cultural values. Finally, the results did not support the hypothesis that generational status moderates these relationships.	Familism ▼ help-seeking attitudes Traditional Gender Roles ▼ help-seeking attitudes	university/ college students, *N* = 350 (21.1%/78.9%/0%) *M* = 24.1 years (18–60 years)	US	cultural	no		x	
Spahlholz et al., (2023)	complementing traditional approaches in help-seeking research by introducing a milieu approach, focusing on values and political attitudes as a possible explanation for differences in help-seeking for emotional mental health problems, with the aim of examining whether self-reported help-seeking behaviour is related to being embedded in a specific milieu	The conclusion of the study is that the likelihood of seeking help for mental health issues varies across different milieus, even when accounting for sociodemographic factors. This effect was more pronounced for seeking psychotherapy than seeking help from a General Practitioner, with the cosmopolitan intellectuals, characterized by liberal ideas and cultural openness, being the most likely group to report seeking help from both psychotherapists and General Practitioners.	cosmopolitan intellectual milieu ▲ help-seeking in history	general population, *N* = 3042 (47.2%/52.4%/0.4%) *M* = 49.2 years	GER	political, milieu-specific	no		x	
Mudryk & Johnson (2023)	exploring how culturally competent care can be integrated for the growing Muslim demographic in the U.S. It addressed two main questions: (1) Do demographics predict reported Islamic religiousness? and (2) to what extent does Islamic religiousness and beliefs about mental health predict treatment preferences?	Analyses found that Islamic religiousness and biomedical beliefs predicted more openness to mental health treatment. Additional analyses found that integration with mainstream culture was correlated with higher confidence in services.	Islamic religiousness ⬧ help-seeking attitudes mediating effect of biomedical etiological beliefs on relationship between Islamic religiousness and attitudes towards mental health services	university/ college students, *N* = 122 (42.6%/54.1%/3.3%) *M* = 32.1 years (18–64 years)	US	religious	no		x	
Plessy et al. (2023)	examining factors influencing African American mothers’ recognition of problematic child behaviour, and exploring how cultural variables affect their intention to use parent training for treatment after identifying a behaviour problem	Among mothers who considered the child as exhibiting problematic behaviour, African American racial identity significantly predicted treatment stigmatization and parent training utilization. This finding indicates that having a stronger centralized racial identity was predictive of higher treatment stigmatization and a lower likelihood of utilizing parent training.	African American racial identity ▼ help-seeking attitudes	Relatives, *N* = 122 (0%/100%/0%) *M* = 32.4 years (20–49 years)	US	cultural	no	x	x	
Schiffer & Saucier (2023)	exploring how masculine honor beliefs (e.g., that aggression is some-times justifiable) and beliefs in mental health stigma relate to the perception of psychological (as compared to physical) military injuries; via both public recognition (such as military honors) and personal support (such as donations to veterans’ organizations	The study found the more strongly one adhered to Military Honor Beliefs, the more strongly they tended to report beliefs in mental health stigma.	masculine honor belief ▲ public & perceived stigma	general population, *N* = 427 Study 1*: n* = 255 (33.0%/64.0%/3.0%) *M* = 45.4 years (19–80 years) Study 2*: n* = 172 (43.0%/55.0%/2.0%) *M* = 48.5 years (21–84)	US	Milieu-specific	no	x	x	
Heller et al. (2024)	testing whether bilingual Chinese-English speakers are more accepting of mental health treatment when presented in English, their second language, compared to their native Chinese	Consistent with a language-priming-culture hypothesis, when using Chinese, the more an individual reported feeling aligned with traditional Asian values, the less they were likely to recommend treatment; however, when using English, their treatment recommendation was independent of their alignment with traditional Asian values.	moderating effect of adherence to traditional Asian values on the relationship between the Chinese language and treatment recommendation	general population, *N* = 323 (27.2%/72.14%/0.6%)	CHI	cultural	no	x	x	
Jones et al. (2024)	exploring how Latina women’s perceptions of their combined ethnic values and gender role attitudes, predict their mental health and attitudes toward help-seeking	Latent Profile Analysis revealed four profiles of Latina women’s ethnocultural gender role (i.e., Integrationist, Separationist, Assimilationist, and Marginalist) that were associated with women’s help-seeking attitudes, but not their mental health. The study showed that stronger affiliation towards traditional Mexican American values was associated with less positive attitudes towards help seeking and pro-feminist and egalitarian attitudes are related to more positive attitudes towards help seeking.	traditional ethnic values ▼ help-seeking attitudes pro-feminist and egalitarian values ▲ help-seeking attitudes	general population, university/ college students, *N* = 257 (0%/100%/0%) *M* = 31.6 years (18–76 years)	US	cultural	no		x	

**Note**. *M* = mean; AUS = Australia, FRA = France, GRE = Greek, UK = United Kingdom of Great Britain, US = United States of America, CAN = Canada, GER = Germany, CHI = China, SVE = sweden, NOR = Norway; f-2-f = face-to-face data collection; f%/m%/d% = fraction in percentage of participants with male/female/other gender in the study sample; LAMIC = Low-and-middle-income countries

## Electronic supplementary material

Below is the link to the electronic supplementary material.

Supplementary material 1

Supplementary material 2

Supplementary material 3

Supplementary material 4

Supplementary material 5

Supplementary Material 6

## Data Availability

No datasets were generated or analysed during the current study.

## Abbreviations

ADHD: Attention Deficit/Hyperactivity Disorder
CM: Colonial Mentality
PRESS: Peer Review of Electronic Search Strategies
PRISMA: Preferred Reporting Items for Systematic Reviews and Meta-Analyses
PRISMA-ScR: PRISMA extension for scoping reviews
UK: United Kingdom of Great Britain
US: United States of America
VASI: Value-based stigma inventory

## Declarations

### Registration for systematic reviews

PROSPERO CRD42020161859
