# Supplementary material for: Values, belief systems and mental health stigma – a scoping review and synthesis of quantitative evidence
Source: BMC Psychiatry. 2026 May 19;26:419. doi: 10.1186/s12888-026-08112-y (PMC13191847; doi:10.1186/s12888-026-08112-y)
Supplement: Supplementary file 1 — Supplementary material 1 [file 12888_2026_8112_MOESM1_ESM.pdf]

PROSPERO
International prospective register of systematic reviews

Personal values and the stigma of mental illness: A systematic review of the literature and meta-analysis of associations among population samples.

Sven Speerforck, Georg Schomerus

To enable PROSPERO to focus on COVID-19 submissions, this registration record has undergone basic automated checks for eligibility and is published exactly as submitted. PROSPERO has never provided peer review, and usual checking by the PROSPERO team does not endorse content. Therefore, automatically published records should be treated as any other PROSPERO registration. Further detail is provided **here**.

Citation

Sven Speerforck, Georg Schomerus. Personal values and the stigma of mental illness: A systematic review of the literature and meta-analysis of associations among population samples.. PROSPERO 2020 CRD42020161859 Available from: https://www.crd.york.ac.uk/prospero/display_record.php?ID=CRD42020161859

Review question

Are specific personal value dimensions associated with stigmatizing attitudes towards people with mental illness?
Are specific personal value dimensions associated with stigmatizing attitudes towards help-seeking for a mental health problem?

Searches

MEDLINE, 1.2.2020
Embase, 1.2.2020
PsycINFO, 1.2.2020
No restrictions to publication period
Language: English

Google Scholar, 1.2.2020
No restrictions to publication period
Language: German

Types of study to be included

All studys (retrospective, case-control, cohort, prospective) will be included that report correlational relationships between values and the 2 outcomes mentionned above. Further publication in a peer-reviewed journal is mandatory.

Condition or domain being studied

Stigma of mental illness, social distance, attitudes, help-seeking, personal values

Participants/population

Inclusion: General Population, Patients, relatives

Intervention(s), exposure(s)

If value measurements are suffciently homogenous for a meta-analysis, reported correlational relationships between transparently operationalized personal values (e.g. Schwartz, Speyer, role-models, single items, "Exposure") and 1) stigma measures (desire for social distance, stereotypes (e.g. dangerousness, blame), emotions (fear, anger) "outcome 1") or 2) attitudes towards help-seeking for a mental health problem "outcome 2"

Comparator(s)/control

Other dimensions of personal values

Context

First we will conduct a systematic review of relevant quantitative studys and give a narrative summary. If value measurements can be suffciently homogenized as a sensible exposure variable a meta-analysis of the gathered data from population studys will be conducted.

Main outcome(s)

Correlational relationships between values and the 2 outcomes mentionned above will be analyzed using spearmans r. standardized Beta of associations between personal values and social distance measure and emotions.

Measures of effect

If value measurements are suffciently homogenous for a meta-analysis, spearman r

Additional outcome(s)

If value measurements are suffciently homogenous for a meta-analysis, standardized Beta of associations between personal values and help-seeking attitudes

Measures of effect

spearman r

Data extraction (selection and coding)

Two researchers will check studies for inclusion (one will screen, other check decisions), disagreement will be solved by discussion involving a third researcher.

Data extraction: year of publication, Study Type, sample, disorder, reported value /stigma measurement, correlation (Spearman), Beta, d if applicable. If value measurements are suffciently homogenous for a meta-analysis, authors will be contacted for missing data. Data will be extracted from two researchers (1 extract, 1 check). Disagreements will be solved by discussion and involving a thrid researcher. A Excel Spreadsheet will be used as documentary and published as supplemental data. A software tool will not be used for data extraction.

Risk of bias (quality) assessment

A risk of bias will be assessed at study level focussing on selection and reporting bias. Two researchers will be involved in quality assessement and disagreement will be resolved with a third researcher.

Strategy for data synthesis

Given a minimum of three studies with a comparable exposure (personal value) on a population level all analysis will be done using Comprehensive Meta-Analysis software relying on correlations using a random effects model. Beta-weights from standardized regression analyses will be converted to correlations using the formula
developed by Peterson and Brown (2005) before being entered into the software. Although beta-weights are adjusted for covariates such as demographic factors Peterson and Brown (2005) argued that using them to impute missing correlations will improve the accuracy and generalizability of the overall effect size estimate.

Analysis of subgroups or subsets

Depending on the number of identified studys (minimum three each), subgroup analysis will be conducted for the respective disorder (mental illness in general, schizophrenia, depression) since stigma levels and influence of values is most likely to differ in this respect

Contact details for further information

Sven Speerforck
sven.speerforck@medizin.uni-leipzig.de

Organisational affiliation of the review

University Leipzig, Department of Psychiatry and Psychotherapy

Review team members and their organisational affiliations

Dr Sven Speerforck. UniversitÃ¤tsklinik fÃ¼r Psychiatrie und Psychotherapie
Professor Georg Schomerus. University of Leipzig, Department of Psychiatry and Psychotherapy

Type and method of review

Narrative synthesis, Systematic review

Anticipated or actual start date

06 January 2020

Anticipated completion date [1 change]

31 December 2024

Funding sources/sponsors

Deutsche Forschungsgemeinschaft, DFG SP 1810/1-1

Conflicts of interest

Language

English

Country

Germany

Stage of review [1 change]

Review Completed not published

Subject index terms status

Subject indexing assigned by CRD

Subject index terms

Humans; Mental Disorders; Social Stigma

Date of registration in PROSPERO

28 April 2020

Date of first submission

10 December 2019

Stage of review at time of this submission [1 change]

Stage	Started	Completed
Preliminary searches	Yes	Yes
Piloting of the study selection process	Yes	Yes
Formal screening of search results against eligibility criteria	Yes	Yes
Data extraction	Yes	Yes
Risk of bias (quality) assessment	Yes	Yes
Data analysis	Yes	Yes

Revision note

just status update approaching publication - thanks

The record owner confirms that the information they have supplied for this submission is accurate and complete and they understand that deliberate provision of inaccurate information or omission of data may be construed as scientific misconduct.

The record owner confirms that they will update the status of the review when it is completed and will add publication details in due course.

Versions

28 April 2020
12 December 2024

PROSPERO
This information has been provided by the named contact for this review. CRD has accepted this information in good faith and registered the review in PROSPERO. The registrant confirms that the information supplied for this submission is accurate and complete. CRD bears no responsibility or liability for the content of this registration record, any associated files or external websites.
