# Supplementary material for: Values, belief systems and mental health stigma – a scoping review and synthesis of quantitative evidence
Source: BMC Psychiatry. 2026 May 19;26:419. doi: 10.1186/s12888-026-08112-y (PMC13191847; doi:10.1186/s12888-026-08112-y)

| stigma/stigmatizing attitude | Stigma* OR stereotyp* OR prejudice* OR Vorurteil* OR "social distance*" OR "soziale Distanz" OR discriminat* OR Diskriminier* OR attitude* OR Einstellung |
| --- | --- |
| values/social milieus | value* OR Wert* OR "social milieu*" OR "soziale* Milieu*" |
| mental illness (general & specific) | "mental illness*" OR "mental disorder*" OR "psychiatric disorder*" OR "psychological disorder*" OR "psychisch* Störung*" OR "psychisch* Krankheit*" OR "mental health" OR "psychisch* Gesundheit" OR "psychisch* Erkrankung*" OR depressi* OR schizophren* OR alcohol* OR "substance use" OR "substance abuse" OR Drogenmissbrauch* OR Substanzmissbrauch* OR bipolar OR "obsessive compulsive" OR Zwang* OR anxiety OR Angst* OR "eating disorder*" OR Essstörung* OR Eßstörung* OR "post-traumatic stress disorder" OR "posttraumatic stress disorder" OR PTSD OR "Posttraumatische Belastungsstörung*" OR PTBS OR psychotic* OR psychotisch* |

## Search algorithm

## Searching Process for EBSCO host

Step 1: Stigma-search algorithm = S1 (TI – Title-search field) & S2 (AB – Abstract search field)

Step 2: value-search algorithm = S3 (TI-field) & S4 (AB-field)

Step 3: mental illness-search algorithm = S5 (TI-field) & S6 (AB-field)
Step 4: connecting the single searches of S1 and S2 to either S1 OR S2 🡪 S7

Step 5: connecting the single searches of S3 and S4 to either S3 OR S4 🡪 S8

Step 6: connecting the single searches of S5 and S6 to either S5 OR S6 🡪 S9

Step 7: connecting all searches: S7 AND S8 AND S9


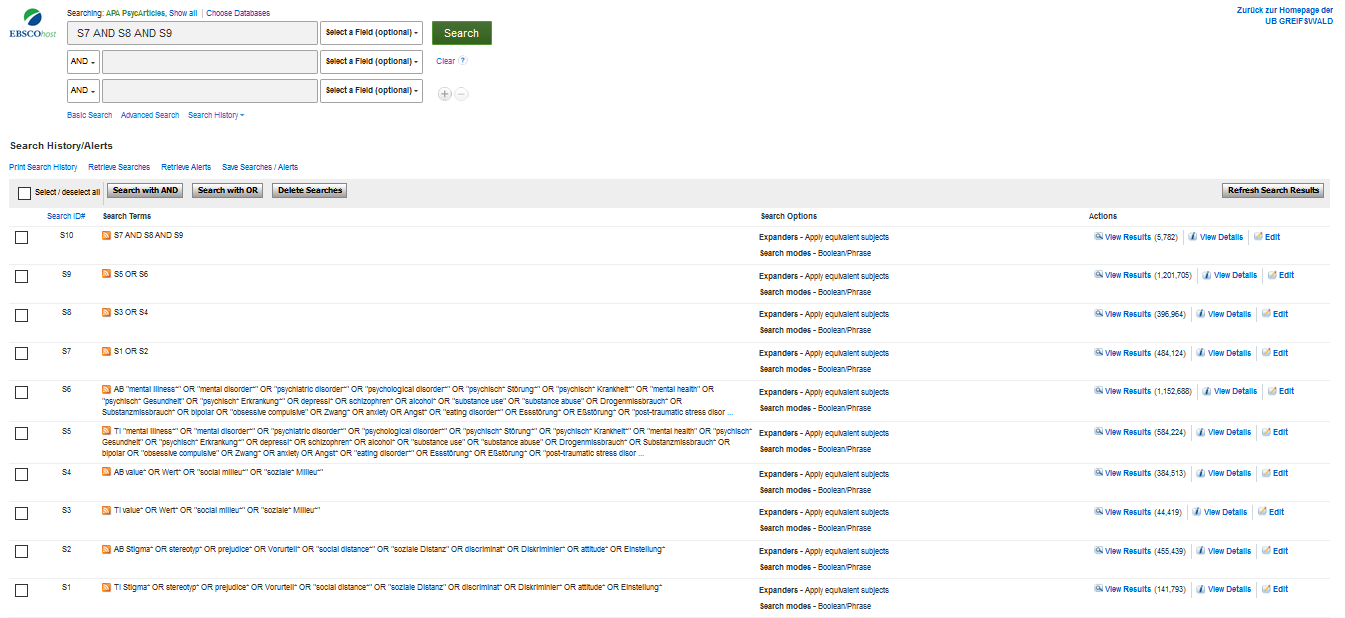


## Searching Process for PubMed (MEDLINE)


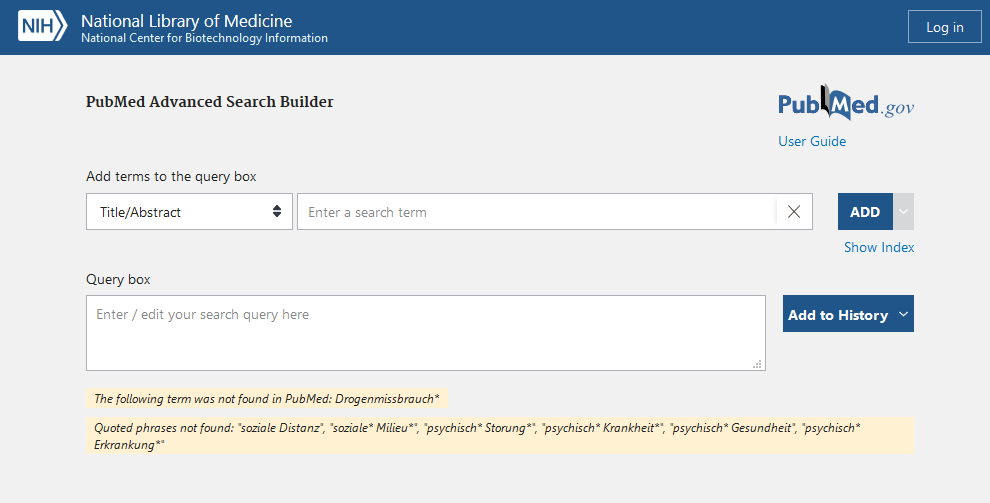


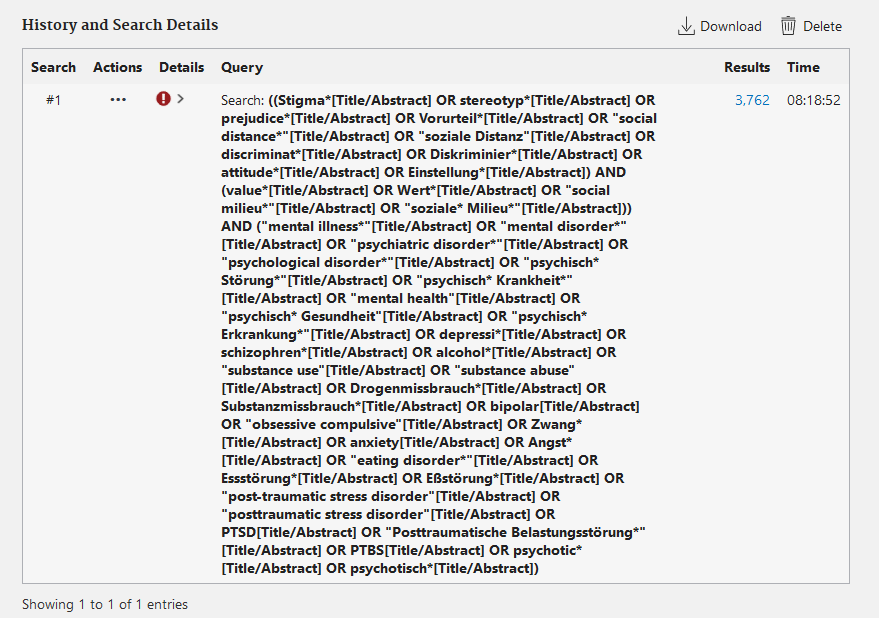

Supplement: Supplementary file 2 — Supplementary material 2 [file 12888_2026_8112_MOESM2_ESM.docx]
