# Supplementary material for: Values, belief systems and mental health stigma – a scoping review and synthesis of quantitative evidence
Source: BMC Psychiatry. 2026 May 19;26:419. doi: 10.1186/s12888-026-08112-y (PMC13191847; doi:10.1186/s12888-026-08112-y)
Supplement: Supplementary file 4 — Supplementary material 4 [file 12888_2026_8112_MOESM4_ESM.docx]

**S4.** Supplementary table. Clustered value dimensions and operationalization.

| Value Dimensions | Operationalization | Σ | References |
| --- | --- | --- | --- |
| Cultural *n* = 22 |  | **31** |  |
|  | Values Survey Module (Werthalung) | 1 | (Hofstede 1984) |
|  | European American Values Scale for Asian Americans (EAVS-AA 18) | 1 | (Wolfe et al. 2001) |
|  | European American Values Scale for Asian Americans – Revised (EAVS-AA-R) 25-item | 3 | Hong, Bryan, Kim , 2006 |
|  | Asian Values Scale (AVS) 36-item | 6 | (Kim et al. 1999) |
|  | Asian Values Scale (AVS-R) 25-item | 3 | (Kim& Hong, 2004) |
|  | Asian American Values Scale-Multidimensional (AAVS-M) 42-item | 1 | (Kim et al. 2005) |
|  | Belief in Chinese Culture and Values Scale (BCCV) 11-item | 1 | (Chen et al. 2008) |
| Individual centrality | | | |
|  | Self-Construal Scale (SCS-25) | 1 | (Singelis) |
|  | Self-Construal Scale (SCS-25), 12-item subscale  interdependence | 1 | (Singelis) |
|  | Vertical-Horizontal Individualism-Collectivism Scale (VHIC, 16-item) | 1 | (Triandis und Gelfand 1998) |
|  | Cultural Orientation Scale (16-item) | 1 | (Triandis und Gelfand 1998) |
| Specific | | | |
|  | Multiphasic Assessment of Cultural Constructs–Short  Form (MACC-SF), 62-true-false-item | 1 | (Israel Cuellar et al. 1995) |
|  | Latino/a Values Scale (LVS -35) | 1 | (Kim et al. 2009) |
|  | Mexican American Cultural Values Scale (MACVS) | 1 | (Knight et al. 2010) |
|  | MACCVS Familism subscales (16 Items from 3 familism subscales), Respect subscale (8 Items) and Traditional gender Roles (5items) from the Mexican American Cultural Values Scale (MACVS) | 1 | (Knight et al. 2010) |
|  | The Multidimensional Inventory of Black Identity (MIBI) Black Centrality 8 Item subscale | 1 | (Robert M. Sellers et al.) |
|  | Loss of Face Concern | 1 | (Nolan Zane and May Yeh) |
|  | Colonial Mentality Scale (CMS) 36-item  For Filipino Americans | 1 | (David und Okazaki 2006) |
| Acculturation Enculturation | | | |
|  | Enculturation Scale for Filipino Americans (ESFA 30-item) | 1 | (Del Prado und Church 2010) |
|  | Acculturation Rating Scale of Mexican Americans–II (ARSMA-II) | 1 | (Israel Cuellar et al.) |
|  | Vancouver Index of Acculturation (VIA -20) | 2 | (Ryder et al. 2000) |
| Religious Spiritual *n* = 6 | | | |
|  |  | **6** |  |
|  | religious and traditional cultural beliefs about depression, 12-item | 1 |  |
|  | Religion subscale from the Mexican American Cultural Values Scale (MACVS), 7-item | 1 |  |
|  | Religiosity Index, 5-items | 1 | Neff& Hope, 1993 |
|  | Religiosity of Islam Scale | 1 | (Jana-Masri & Priester, 2007) |
|  | Religious Beliefs about MI -16 | 1 | (Wesselmann & Graziano, 2010) |
|  | The Christian Orthodoxy Scale | 1 | (Hunsberger, 1989) |
|  | | | |
| Political *n* = 8 |  | **16** |  |
|  | political ideology self reported political (party) affiliation | 2 | (Löve et al. 2019; DeLuca et al. 2018) |
|  | Political Attitudes Scale (28 items) | 1 | Müller-Hilmer, R., & Gagné, J. (2018) |
| Belief Systems |  |  |  |
|  | Belief in a Just World (BJW) | 1 | Dalbert |
|  | Belief in a Just World Scale – 16 items  Self, other subscales | 1 | Lipkus 1991 |
|  | Competitive World Beliefs (CWB) | 1 | Perry &Sibley, 2013 |
|  | Protestant Ethic Scale 11-items | 1 | Katz&Hass, 1988 |
|  | Aspiration Index (AI) | 1 | Kasser& Ryan, 1993 |
| Social Dominance and Authoritarism | | | |
|  | Social Dominance Orientation (SDO) – 12 items | 2 | Pratto, et al, 1994 |
|  | 4-Item Version of SDO | 1 | Duarte, et al, 2004 |
|  | Right-Wing Authoritarianism (RWA) Scale – 20 items | 2 | Altemeyer, 1981 |
|  | 10-Item Version of RWA | 1 | Dru, 2003 |
|  | Right-Wing Extremist Orientation (Rechtsextreme Orientierung) | 2 | Brähler& Faller, 1993 |
|  |  |  |  |
| Personal *n* = 12 |  | **8** |  |
|  | NEOFFI – Openness to Experience-12 | 1 | (McCrae &Costa, 1992) |
|  | Schwartz Value Survey, 57-item | 3 | (Schwartz. 1992) |
|  | Portrait Value Questionnaire (PVQ), 57-items | 2 | (Schwartz &Bardi, 2001) |
|  | 18-item behavioral pattern list | 1 | (Maag, 1989) |
|  | value orientation scale (18-item) | 1 | (Müller_Hilmer&Gangné, 2018) |
| Milieu- specific *n* = 8 | | **5** |  |
|  | Liverpool Stoicism Scale (LSS), 20-item | 1 | Hagger & Chatzisarantis (2009) |
|  | Masculine Honor Beliefs Scale (MHBS) 1LAMIC | 1 | Mahalingam & Kelsay, (2006) |
|  | Honor Ideology of Manhood (HIM-16) | 1 | Barnes , 2012 |
|  | subjective norms 10-items | 1 | (Hammer&Vogel, 2013) |
|  | Composite “suburban values” (dense+cons) | 1 | (Gonzales et al. 2018) |
|  |  |  |  |

| **Value Cluster** | Tendency more stigma | Tendency less stigma |
| --- | --- | --- |
| **Cultural** | Asian Values (collectivsitic), *face concern* | Eur.American Values (individualistic), |
| **Spiritual** | Religious fundamentalism; Evangelical christians (more causal belief: sins, spiritual causes); traditional religious beliefs (“recitation from Koran”) | no association: christian orthodoxy; (in traditional religious muslim community) : strong emotional support from family |
| **Political** | More: Right-wing authoritarianism, social dominance orientation, conservative | More: liberal, egalitarian, “modern” |
| **Personal** | Self-enhancement | Self-transcendence |
| **Milieu-specific** | „stoicism“, „agriarian values“, honor ideology of manhood (despite gender); “suburban”=density of neighborhood+more conservatism); stigmatizing family attitudes (+labeling practice) | Protective family factors |

**References**

Altemeyer, B. (1981). *Right-wing authoritarianism*. Univ. of Manitoba Press.

Chen, Sylvia Xiaohua; Benet-Martínez, Verónica; Harris Bond, Michael (2008): Bicultural identity, bilingualism, and psychological adjustment in multicultural societies: immigration-based and globalization-based acculturation. In: *Journal of personality* 76 (4), S. 803–838. DOI: 10.1111/j.1467-6494.2008.00505.x.

David, E. J. R.; Okazaki, Sumie (2006): Colonial mentality: a review and recommendation for Filipino American psychology. In: *Cultural diversity & ethnic minority psychology* 12 (1), S. 1–16. DOI: 10.1037/1099-9809.12.1.1.

Del Prado, Alicia M.; Church, A. Timothy (2010): Development and validation of the Enculturation Scale for Filipino Americans. In: *Journal of counseling psychology* 57 (4), S. 469–483. DOI: 10.1037/a0020940.

DeLuca, Joseph S.; Vaccaro, John; Seda, Jenna; Yanos, Philip T. (2018): Political attitudes as predictors of the multiple dimensions of mental health stigma. In: *The International journal of social psychiatry* 64 (5), S. 459–469. DOI: 10.1177/0020764018776335.

Gonzales, Lauren; Yanos, Philip T.; Stefancic, Ana; Alexander, Mary Jane; Harney-Delehanty, Brianna (2018): The Role of Neighborhood Factors and Community Stigma in Predicting Community Participation Among Persons With Psychiatric Disabilities. In: *Psychiatric services (Washington, D.C.)* 69 (1), S. 76–83. DOI: 10.1176/appi.ps.201700165.

Hofstede, G. (Hg.) (1984): Culture's consequences: International differences in work-related values (Vol. 5).: sage.

Israel Cuellar; Bill Arnold; and Roberto Maldonado: Acculturation Rating Scale for Mexican Americans-II: A Revision of the Original ARSMA Scale.

Israel Cuellar; Bill Arnold; and Roberto Maldonado (1995): Acculturation Rating Scale for Mexican Americans-II: A Revision of the Original ARSMA Scale.

Kim, Bryan S. K.; Atkinson, Donald R.; Yang, Peggy H. (1999): The Asian Values Scale: Development, factor analysis, validation, and reliability. In: *Journal of counseling psychology* 46 (3), S. 342–352. DOI: 10.1037/0022-0167.46.3.342.

Kim, Bryan S. K.; Li, Lisa C.; Ng, Gladys F. (2005): PsycTESTS Dataset.

Kim, Bryan S. K.; Soliz, Alicia; Orellana, Blanca; Alamilla, Saul G. (2009): Latino/a Values Scale. In: *Measurement and Evaluation in Counseling and Development* 42 (2), S. 71–91. DOI: 10.1177/0748175609336861.

Knight, George P.; Gonzales, Nancy A.; Saenz, Delia S.; Bonds, Darya D.; Germán, Miguelina; Deardorff, Julianna et al. (2010): The Mexican American Cultural Values scales for Adolescents and Adults. In: *The Journal of early adolescence* 30 (3), S. 444–481. DOI: 10.1177/0272431609338178.

Löve, Jesper; Bertilsson, Monica; Martinsson, Johan; Wängnerud, Lena; Hensing, Gunnel (2019): Political Ideology and Stigmatizing Attitudes Toward Depression: The Swedish Case. In: *International journal of health policy and management* 8 (6), S. 365–374. DOI: 10.15171/ijhpm.2019.15.

Nolan Zane and May Yeh (Hg.): THE USE OF CULTURALLY-BASED VARIABLES IN ASSESSMENT: STUDIES ON LOSS OF FACE. Chapter 9.

Robert M. Sellers; Mia A. Smith; J. Nicole Shelton; Stephanie A.J. Rowley; and Tabbye M. Chavous: Multidimensional Model of Racial Identity: A Reconceptualization of African American Racial Identity.

Ryder, A.; Lynn, E. A.; Delroy, L. (2000): Is Acculturation Unidimensional or Bidimensional? A Head-to-Head Comparison in the Prediction of Personality, Self-Identity, and Adjustment.

Singelis, Theodore M.: The Measurement of Independent and Interdependent Self-Construals.

Triandis, Harry C.; Gelfand, Michele J. (1998): Converging measurement of horizontal and vertical individualism and collectivism. In: *Journal of Personality and Social Psychology* 74 (1), S. 118–128. DOI: 10.1037/0022-3514.74.1.118.

Wolfe, M. M.; Yang, P. H.; Wong, E. C.; Atkinson, D. R. (2001): Design and development of the European American values scale for Asian Americans. In: *Cultural diversity & ethnic minority psychology* 7 (3), S. 274–283. DOI: 10.1037/1099-9809.7.3.274.
